# The impact of climate change on western *Plethodon* salamanders’ distribution

**DOI:** 10.1002/ece3.7735

**Published:** 2021-06-29

**Authors:** Sir Nottingham, Tara A. Pelletier

**Affiliations:** ^1^ Department of Biology Radford University Radford VA USA

**Keywords:** climate change, conservation, Pacific Northwest, phylogenetic diversity, *Plethodon*, species distribution model

## Abstract

**Aim:**

Given that salamanders have experienced large shifts in their distributions over time, we determined how each species of *Plethodon* in the Pacific Northwest would respond to climate change. We incorporated several greenhouse scenarios both on a species‐by‐species basis, and also using phylogenetic groups, with the aim to determine the best course of action in managing land area to conserve diversity in this group.

**Location:**

Pacific Northwest of the United States (northern CA, OR, WA, ID, and MT).

**Major taxa studied:**

Western *Plethodon* salamanders.

**Methods:**

Species distribution models were estimated using MaxEnt for the current time period and for several future climate scenarios using bioclimatic data layers. We used several methods to quantify the change in habitat suitability over time from the models. We explored aspects of the climate layers to determine whether we can expect a concerted response to climate change due to similarity in ecological niche or independent responses that could be harder to manage.

**Results:**

The distribution of western *Plethodon* salamander species is strongly influenced by precipitation and less so by temperature. Species responses to climate change resulted in both increases and decreases in predicted suitable habitat, though most species ranges do not contract, especially when taken as a phylogenetic group.

**Main conclusions:**

While some established habitats may become more or less climatically suitable, the overall distribution of species in this group is unlikely to be significantly affected. Clades of *Plethodon* species are unlikely to be in danger of extirpation despite the possibility that individual species may be threatened as a result of limited distributions. Grouping species into lineages with similar geographic ranges can be a viable method of determining conservation needs. More biotic and dispersal information is needed to determine the true impact that changes in climate will have on the distribution of *Plethodon* species.

## Graphical abstract

We determined how each species of terrestrial salamander (*Plethodon*) from the Pacific Northwest would respond to climate change under several greenhouse scenarios. We asked whether we can expect a concerted response to climate change due to similarity in ecological niche or independent responses that could be harder to manage across species. While some established habitats may become more or less suitable over time, the overall distribution of species in this group is unlikely to be significantly affected, and distributions in this group are more strongly influenced by precipitation than temperature.

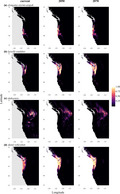

## 1 INTRODUCTION

Species range distributions shift over evolutionary timescales, both expanding and contracting within the cyclical nature of glaciations and periods of warmth (Pielou, 1991). Given that we are seeing a rapid increase in global temperatures, we might expect species ranges to shift accordingly at a much faster rate than has been observed in the past; this will be even more challenging for species living in mountainous regions, such as the Pacific Northwest (PNW) of the United States (Dobrowski & Parks, 2016). Many species are predicted to go extinct, though how to predict which species are at the highest risk is still unclear (Urban, 2015). Even similar species may have unique responses to similar environmental changes (Rapacciuolo et al., 2014), and in some cases, especially when there are rapid changes to the environment, it will be necessary for species to traverse into new territory, rather than adapt (Bridle & Vines, 2007). Salamanders may be particularly at risk because temperate amphibians are highly vulnerable to changes in temperature (Gerick et al., 2014), while also having limited dispersal capabilities (Ovaska, 1988; Smith & Green, 2005).

Endemic species with small distributions are particularly prone to extinction (Schwartz et al., 2006); therefore, it is important to understand the habitat requirements of such species, as well as estimate the potential for range contractions under future climate scenarios. Species distribution models (SDMs) use locality data from throughout a species distribution in conjunction with layers of climate data to predict the environmental envelope of that species, which can then be projected onto a geographic map (Peterson, 2006; Soberon & Peterson, 2005). Species occurrence data are used to extract environmental information about environmental factors that represent suitable areas for a species to exist. These models are often used to predict current distributions (Sarquis et al., 2018), understand environmental envelopes (Manzoor et al., 2020), and predict changes in species distributions from the past (Ding & Liao, 2019) and into the future (Zhang et al., 2020).

Current geographic distributions certainly play a role in a species response to climate change and an understanding of the limitations of species will help predict how easily that species may disperse into new territory (Peterman & Semlitsch, 2014). Furthermore, projecting the environmental envelope of a species onto future climate models has been used to assess the available habitat and dispersal corridors under climate change for conservation purposes (Esser et al., 2019; Zellmer et al., 2020; Zhang et al., 2019). If a species suitable habitat shrinks with changing conditions, as we expect to be the case in salamanders, they may be at higher risk of extinction (Thomas et al., 2004). For example, Jacobsen et al. (2020) used SDMs to assess the relationship between landscape features and the environment to locations in which *P. punctatus*, an eastern Plethodontid, can be found. They found that most of this species climatic niche will be gone by 2,100. However, the amount of habitat loss can be species‐dependent in salamanders (Sutton et al., 2015).

**FIGURE 1:**
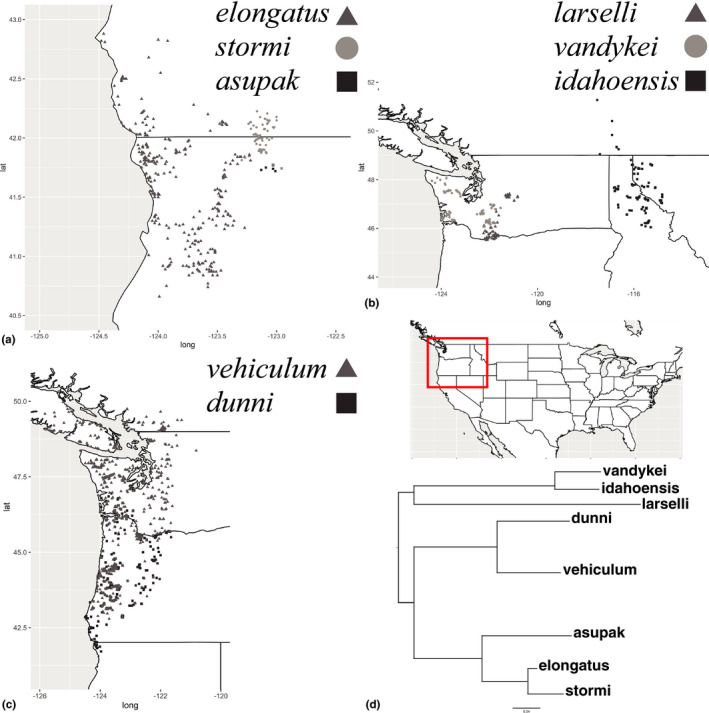
Species localities and phylogenetic relationship based on mtDNA. Localities for each species are representative of the distribution for each species. (a) Localities for *Plethodon asupak*, *elongatus*, and *stormi* in CA and OR. (b) Localities for *Plethodon idahoensis*, *larselli*, and *vandykei* in OR, WA, ID, MT, and BC. (c) Localities for *Plethodon dunni* and *vehiculum* in OR, WA, and BC. (d) Phylogenetic tree for all PNW *Plethodon* species. Only the species tips are shown here because all species were monophyletic. For the full tree, see Figure S25

Eight *Plethodon* salamanders reside in the PNW. Plethodontid salamanders are fully terrestrial and lungless; they rely on cool moist environments for survival and are found in forested areas, often close to the splash zones of streams and/or the runoff from melting snow fields (Corkran & Thoms, 2006). Plethodontid salamanders are generally considered dispersal limited, though there is some indication that they can travel far distances (Marsh et al., 2004; Ovaska, 1988; Smith & Green, 2005). The PNW has a complex geologic history and landscape features (Brunsfeld et al., 2001), resulting in highly divergent distribution patterns among these species (Figure 1). Many species occur in sympatry, unlike their eastern counterparts (Kozak & Wiens, 2006), sometimes demonstrating extensive overlap in their ranges, and there is large variation in the size of their distributions. Range expansion has been a common pattern in this group, along with other salamanders in the region, since the Pleistocene glaciations, and rivers only seem to inhibit dispersal in some species (Carstens et al., 2004; Kuchta & Tan, 2005; Mahoney, 2004; Miller et al., 2005, 2006; Steele & Storfer, 2006, 2007; Wagner et al., 2005, 2006). Even though Plethodontids appear morphologically and ecologically constrained (Mueller et al., 2004; Wake, 2009), they sometimes inhabit distinct environmental niches and/or respond to changes in the climate in unique ways (Pelletier & Carstens, 2016; Pelletier et al., 2015). These unique patterns make the role that climate change will play in the future of these species difficult to predict. There are 3 main lineages in this group (Figure 1), and each will be discussed below. We note that there is one other western *Plethodon* species that we are not considering here due to its distribution not being located in the PNW: *P*. *neomexicanus* (Stebbins & Riemer, 1950).

*Plethodon asupak* (Mead et al., 2005), *elongatus* (Van Denburgh, 1916), and *stormi* (Highton & Brame, 1965): These three sister species occur in southern Oregon (OR) and northern California (CA). They all display high levels of genetic variation within and among populations, with *P*. *elongatus* having the largest distribution of these species. Three genetic populations have been observed in *P. elongatus* and only the north western group shows evidence of range expansion (Mahoney, 2004). *Plethodon stormi* is restricted to the Siskiyou Mountains and overlaps the larger distribution of *P. elongatus*. It has two distinct genetic populations, but rivers do not seem to act as a barrier to dispersal (Mead et al., 2005). Similarly, *P. asupak* has a small distribution, the most restricted range of any western *Plethodon*, and is also parapatric with *P. elongatus* (Mead et al., 2005).

*Plethodon idahoensis* (Slater & Slipp, 1940), *larselli* (Burns, 1962), and *vandykei* (Van Denburgh, 1906): These three sister species have striking differences in their distributions and diverged from each other approximately 5mya (Kozak et al., 2009). *Plethodon idahoensis* occupies a large, continuous range from central Idaho (ID) to southern British Columbia (BC). This is the only *Plethodon* species found east of the Columbia Basin in the Northern Rocky Mountains. Extensive northern population expansion since the Pleistocene has been detected and there does appear to be some population structure that separates the northern and southern river drainages, likely a remnant of glacial refugia during the last glacial maximum (LGM) (Carstens et al., 2004, 2005, 2009; Carstens & Richards, 2007; Pelletier & Carstens, 2014). Moreover, these two populations represent two different evolutionary lineages that display environmental niche differentiation (Pelletier et al., 2015), which could influence species and/or conservation status going forward, especially if population sizes and geographic ranges shrink with the changing climate. On the other hand, *P. vandykei* occupies three small, disjunct ranges in Washington (WA). However, data suggest these populations have not been isolated for very long, as they show little genetic differentiation, large amounts of suitable habitat between populations during the LGM, and similar ecological niches across regions (Pelletier et al., 2015). *Plethodon larselli* also has three small, somewhat disjunct ranges in OR and WA where migration among regions seems to be limited, partially due to rivers and high elevation mountains (Wagner et al., 2005). This species has stricter habitat requirements than the other species (Aubry et al., 1987; Pelletier et al., 2015). Similar to *P. vandykei*, *P. larselli* might have been more connected during the LGM and might even be currently dispersing across the Columbia River (Pelletier et al., 2015).

*Plethodon dunni* (Bishop, 1934) and *vehiculum* (Cooper, 1860): Both of these sister species have large continuous distributions in OR and WA that cross the Columbia River, with some population genetic structure (Pelletier & Carstens, 2016). They are largely sympatric and diverged from one another approximately 10 mya (Kozak et al., 2009). These two species had similar southern ancestral distributions during the LGM, and there is evidence of extensive range expansion in both species, yet *P. vehiculum* was able to expand much farther north than *P. dunni* in the last 20,000 years, even though the effective population size of *P. dunni* grew faster (Pelletier & Carstens, 2016). Overall, *P*. *vehiculum* has the largest range with very little genetic structure, even onto Vancouver Island (Pelletier et al., 2011), and has a higher tolerance to extremes in temperature and humidity (Dumas, 1956), indicating this species is a good disperser.

In this study, we have several aims. First, we estimate the species distribution model for each species using climatic data layers. We compare the environmental limitations across species with the expectation that each species will have a unique environmental envelope. This can be particularly useful because it is possible that not all species will respond to climate change similarly (Pucko et al., 2011; Zhang et al., 2019). Next, we project the current species environmental envelope onto future climate models to gain information about where these species may shift their ranges to overcome the increase in global temperature as this information may inform land‐use policy (Brown & Yoder, 2015; Wilting et al., 2010). In addition to estimating current and future SDMs for each species, we estimate models for clades that include 2–3 closely related species as this can improve niche estimation by taking into account the shared environmental tolerances among evolutionary lineages (Smith et al., 2018). Biodiversity can be defined at multiple levels (populations, species, communities) and by considering conservation at more than one level (i.e., not only at the species level), we may come up with conservation strategies that are easier to implement (Marcot, 2006). Efforts aimed at conserving phylogenetic diversity rather than focusing on individual species (Faith, 1992) can offer more manageable conservation solutions in defining protected areas (Rosauer et al., 2018).

## 2 METHODS

### 2.1 Phylogenetic tree

We estimated a phylogenetic tree to lend support to the potential of estimating SDMs using phylogenetic groups, and there has yet to be a single phylogenetic tree that includes every western *Plethodon* species. We used the mitochondrial gene *cytochrome b* (cytb) by downloading sequences from GenBank for *P. asupak* (*n* = 6), *P. elongatus* (*n* = 113), and *P. stormi* (*n* = 37) that were between 300 and 400 base pairs because this region of cytb was the most well‐represented (accession numbers in Table S8). We used data from Pelletier et al. (2015) for *P. idahoensis* (*n* = 21), *P. larselli* (*n* = 18), and *P. vandykei* (*n* = 18), and data from Pelletier and Carstens (2016) for *P. dunni* (*n* = 116) and *P. vehiculum* (*n* = 184). MAFFT v7.453 (Katoh & Standley, 2013) was used to align the sequences which were then checked by eye in Mesquite v3.6 (Maddison & Maddison, 2019). Sequences were trimmed to remove large amounts of missing data, and we were left with 385bp in the sequence alignment. jModelTest v.2.1.10 (Darriba et al., 2012) was used to determine the model of sequence evolution. We estimated the species tree in BEAST v1.10.4 (Suchard et al., 2018). Default parameters from BEAUTI v1.10.4 were used, except for the model of sequence evolution determined by jModelTest (TVM + I + G). Trial runs were conducted to assess the default settings. Two independent runs were conducted for 20,000,000 generations each, sampling every 2000 steps, with 10% removed for burn‐in. Convergence and ESS values were confirmed using Tracer v1.7.1 (Rambaut et al., 2018), and runs were combined (LogCombiner v1.10.4) to estimate the maximum clade credibility tree (TreeAnnotator v1.10.4). We estimated species distribution models for each species and each species group based on their phylogenetic relatedness (see results), totaling 8 species (*asupak*, *elongatus*, *stormi*, *larselli*, *vandykei*, *idahoensis*, *dunni*, and *vehiculum*) and 4 groups (*asupak* + *elongatus* + *stormi*, *larselli* + *vandykei*, *larselli* + *vandykei* + *idahoensis*, and *dunni* + *vehiculum*). We chose to assess models from both *larselli* + *vandykei* and *larselli* + *vandykei* + *idahoensis* because while these three species are closely related, *P. idahoensis* does not share a geographic range similar to any of the other species.

### 2.2 Data

We downloaded species occurrence data from GBIF on a species‐by‐species basis (DOIs reported in Table 1). Only those with GPS coordinates and no known issues from human observation, material sample, observation, and preserved specimen were retained. Additional GPS coordinates for *P*. *idahoensis, larselli,* and *vandykei* were taken from Pelletier et al. (2015), and iDigBio for *P. asupak, idahoensis, larselli, stormi, and vandykei*. Once all GPS coordinates were collected, several cleaning steps were applied to prevent sampling bias and incorrect data points (Table 1). All data manipulation and analyses were conducted using R v3.6.1 (R Core Team, 2019).

**TABLE 1:** IUCN Red list category for each species, the number of localities before data cleaning steps (*n*_0_), the number of localities used for the analyses after data cleaning steps and extracting one point per grid cell (*n*), the number of unique GPS coordinates found within some sort of protected area (*n*_p_), and DOIs for all downloads

*Plethodon*	IUCN listing	*n*_0_	*n*	*n*_p_	GBIF DOI	iDigBio DOI
*asupak*	VU	39	6	4	https://doi.org/10.15468/dl.3malaj	http://s.idigbio.org/idigbio‐downloads/85a48039‐a9ca‐4825‐b022‐661dedff34c1.zip
*dunni*	LC	436	299	30	https://doi.org/10.15468/dl.qzbaiy	NA
*elongatus*	NT	311	275	112	https://doi.org/10.15468/dl.vtvha8	NA
*idahoensis*	LC	126	112	8	https://doi.org/10.15468/dl.ysrazd	http://s.idigbio.org/idigbio‐downloads/1e07950a‐fe77‐4f9d‐bb30‐8abc539e0f99.zip
*larselli*	NT	213	76	22	https://doi.org/10.15468/dl.zsftzi	http://s.idigbio.org/idigbio‐downloads/aa934cd3‐3bb4‐4e0c‐a019‐4dfc91317878.zip
*stormi*	EN	244	63	8	https://doi.org/10.15468/dl.rvq1jv	http://s.idigbio.org/idigbio‐downloads/85a48039‐a9ca‐4825‐b022‐661dedff34c1.zip
*vandykei*	LC	317	109	133	https://doi.org/10.15468/dl.nst81m	http://s.idigbio.org/idigbio‐downloads/1e07950a‐fe77‐4f9d‐bb30‐8abc539e0f99.zip
*vehiculum*	LC	913	664	234	https://doi.org/10.15468/dl.2dbr0s	NA

We standardized the number of decimal places to three for the designation of latitude and longitude followed by removing duplicate coordinates. In order to verify the accuracy of the data points, shape files that represent the species known distributions were acquired from IUCN Red List (https://www.iucnredlist.org/). For each species, location samples were plotted on corresponding IUCN shape files using the package *ggplot2* (Wickham, 2016). Points falling outside the established area were considered suspect and reviewed further. Many suspect points were removed from each species in order to avoid skewing the results due to potentially improperly identified samples. For each species, specific criteria were used to determine which points outside of the IUCN distribution should be removed, based on known habitat information regarding the species (see Figures S1–S8 descriptions for details). After removal of localities within the same 1 km grid cell to prevent pseudo‐replication, a total of 6 *P. asupak*, 299 *P. dunni*, 275 *P. elongatus*, 112 *P. idahoensis*, 76 *P. larselli*, 63 *P. stormi*, 109 *P. vandykei*, and 664 *P. vehiculum* were used for modeling (Table 1).

Climate data were sourced from WorldClim v1.4 (https://www.worldclim.org/) at 30 arc‐second resolution (1 km; Hijmans et al., 2005; Fick & Hijmans, 2017). All climate layers were cropped to include the relevant geographic space of the PNW for all species using the *raster* package (Hijmans, 2019). The boundary was 37.00 to 55.00 latitude, and −130.00 to −113.00 longitude in order to encompass all species distributions for comparison, leave room for expansion, but limit completely unlimited dispersal. Data include the 19 bioclimatic variables averaged from 1970–2000. We conducted pairwise Pearson correlations for all variables for each species by extracting the values for each variable at each GPS coordinate (Table S1). We removed those that were highly correlated (>0.9 and <−0.9) for each species/group that was modeled.

### 2.3 Species distribution modeling

Models were estimated in two ways for each species/group: (1) using bioclimatic variables after removing those that were highly correlated, and (2) using a full set of 19 bioclimatic variables, plus elevation, and two summary variables (mean and standard deviation [*SD*]) for solar radiation, wind speed, and water vapor pressure, totaling 26 variables. For this second set of models, we included all variables so that the environmental variables could be more directly compared across species and excluding highly correlated variables has been shown to not significantly improve model performance when using MaxEnt (Feng et al., 2019). We estimated the SDMs using MaxEnt v3.4.1 (Phillips et al., 2017), which applies a machine‐learning technique known as maximum entropy (Elith et al., 2011). We implemented MaxEnt via the R package *ENMeval* and tested model fit using AIC (the Akaike Information Criterion) and several evaluation statistics for 18 combinations of modeling parameters (Muscarella et al., 2014). AUC_test_ is the area under the receiver operating characteristic curve, which measures the performance of the model against actual observations, where values closer to 1 are better. AUC_diff_ measures the difference between test and training data where a higher value may indicate model over‐fitting. OR_MTP_ and OR_10_ are threshold‐dependent tests that measure the proportion of test localities against suitability values lower than the lowest‐ranking training localities, where lower scores are better.

We used the default random test percentage of 25 and sampled 10,000 random background test points. Jackknife partitioning was used for species/groups with a continuous distribution while checkerboard1 partitioning was used to model species/groups with several disjunct distributions (Muscarella et al., 2014). For each species/group, we ran MaxEnt models using several combinations of feature class (L, LQ, H, LQH, LQHP, LQHPT), which determine how predictor variables are transformed, where L = linear, Q = quadratic, H = hinge, P = product, and T = threshold. We also tested three regularization multipliers (1, 2, and 3) for each feature class that was modeled, totaling 18 models per species/group. The best model was chosen using AICc for all following analyses. Using the R package *ENMTools* (Warren & Dinnage, 2020; Warren et al., 2009), we calculated the niche breadth on the predictions of habitat suitability (Nakazato et al., 2010) for each species/group, where values closer to 0 mean a species has a low niche breadth based on the environmental variables used. Then, we calculated how much the projections across species overlapped (*D*; Schoener, 1968), where 0 means no overlap and 1 means identical predictions.

We used future climate layers available on WorldClim v1.4 at 30 arc‐second resolution to project the species distribution models using several future climate scenarios. Layers were treated as those above. The climate layers used for modeling were derived from the algorithms CCSM4 (Vertenstein et al., 2020) and MIROC5 (Tatebe et al., 2012) to include models with different levels of complexity (Mehta et al., 2013) for the years 2050 (averaged 2041–2060) and 2070 (averaged 2061–2080). We included greenhouse gas scenarios rcp4.5 (an intermediate scenario where emissions peak around 2040 but decline) and rcp8.5 (a worst‐case scenario where emissions continue to increase), to identify changes from least to most extreme, resulting in 4 future climate models for both algorithms. We averaged the models for each year (2050 and 2070) so that four models contributed to the average: CCSM4 and MIROC5 and rcp4.5 and rcp8.5 to create mean projections for visualization and some analyses below.

We assessed how the SDMs changed over time in several different ways. First, we compared projection overlap from the current model to those for each future projection (8 per species/group) using the R package *ENMTools* as above. Next, we calculated the sum and mean of the raster layer for each SDM (i.e., the suitability scores for each model were summed and averaged) in order to quantify suitability of available habitat. The current value was subtracted from the future value to report a single number that represents change for each future model (8 per species/group). In this case, a negative number represents a reduction in suitability from the current model into the future, while a positive number represents an increase in suitability from the current model into the future. Finally, we wanted to estimate change in the SDMs only for grid cells with higher suitability scores and therefore more likely to harbor individuals. For the current model and each mean future model (2 years per species/group), threshold suitability scores were set at 0.6 and 0.8, where all grid cells with a suitability score above the threshold were counted. The current value was subtracted from the future value for each species/group. A negative number represents a reduction in suitability from the current model into the future, while a positive number represents an increase in suitability from the current model into the future.

In order to determine the impact of each climatic variable for each species, climatic data were extracted using the GPS coordinates for each species, for all bioclimatic variables, at both the current time and all future climate scenarios. A *t*‐test was conducted to determine if there was a significant difference between the current and future values, at the locations in which the species is currently located. A Bonferroni correction was applied to assess significance; for each species/group, there were 19 variables for 8 future models (*α* = 0.05/(19*8) = 0.0003).

## 3 RESULTS

### 3.1 Summary

**FIGURE 2:**
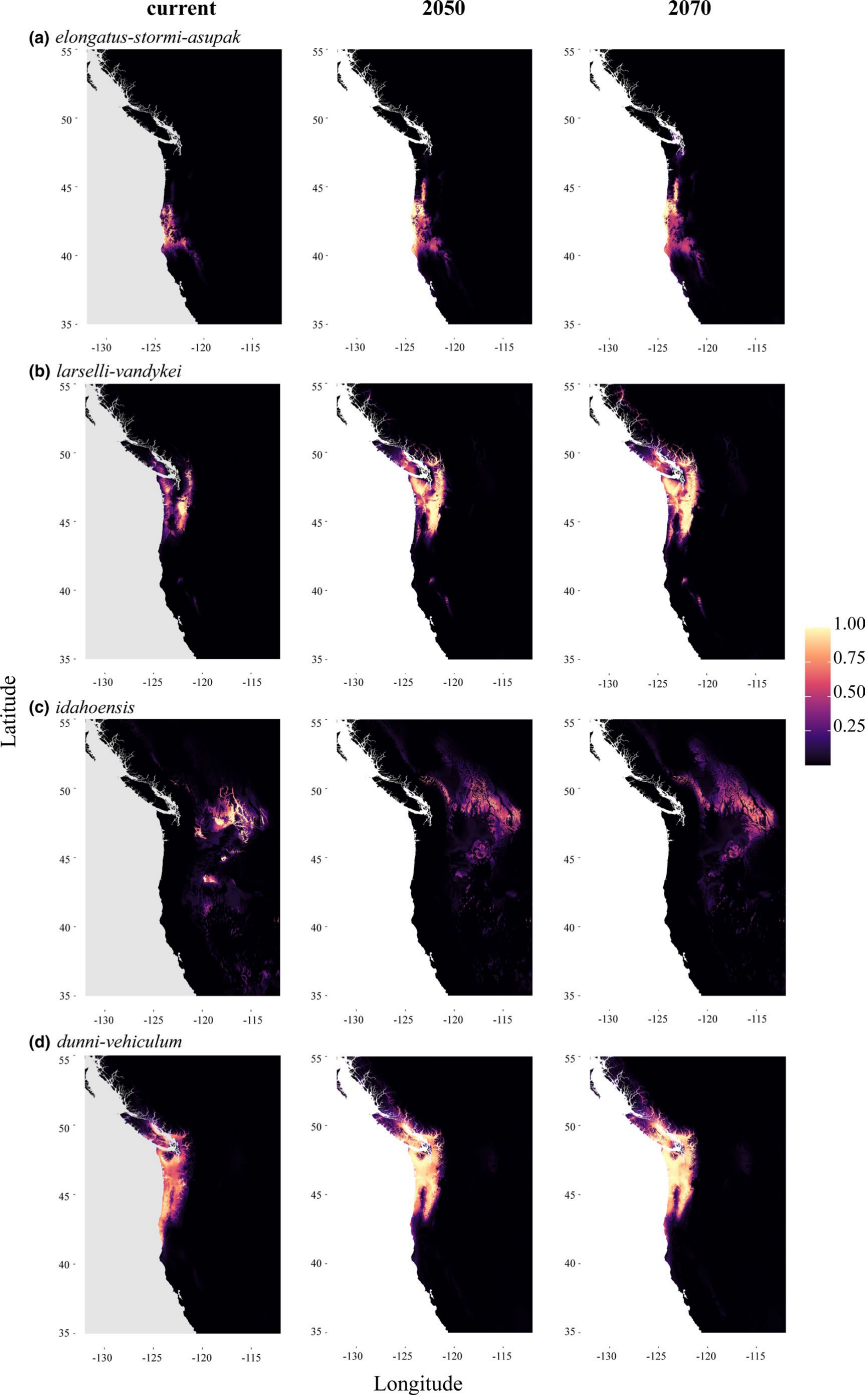
Current and future species distribution models. (a) *P. elongatus‐stormi‐asupak*. (b) *P. larselli‐vandykei*. (c) *P. idahoensis*. (d) *P. dunni‐vehiculum*. The future models depicted here are averaged by year and include CCSM4 and MIROC5 models and both the rpc4.5 and rpc8.5 climate scenarios. All other current and future models can be found as Figures S9–S24

The phylogenetic tree analysis was consistent with previous investigations on the evolutionary history of western *Plethodon* salamanders, and all species were monophyletic, except one *P. asupak* individual nested within *P. stormi*, which we suspect is a likely misidentification (Figure 1d and S25; Kozak et al., 2009). This analysis is the first phylogenetic tree to include all PNW *Plethodon* species and supports our phylogenetic groups used for modeling. There were over at least 76 GPS coordinates for all species except *P. asupak* for which we were only able to locate 6 unique GPS coordinates (Table 1). We include the results for *P. asupak* with the caveat that these results should be interpreted with caution and are therefore not discussed in any detail. For the remainder of the results, we focus on models that had the lowest AICc score from our model evaluation test (Tables 2 and S2). All AUC_test_ scores were above 0.96, while most were above 0.98, and all AUC_diff_ scores were low, with the exception of *P. asupak*. AUC_test_ was generally higher for the single‐species models over the group models (Table 2). All OR scores were low, with the exception of *P. asupak*. The current SDMs match what is known about the current distribution for all species/groups, with the exception of *P. asupak*. We display models for the *P. elongatus‐stormi‐asupak*, *P. larselli‐vandykei*, and *P. dunni‐vehiculum* groups because these represent closely related lineages that have a large overlap in their ranges, and *P. idahoensis* on its own because it is the only species found east of the Columbia Basin (Figure 2). All projections can be found as Figures S9–S16. Models using the full set of variables are presented as Figures S17–S24 and do not differ substantially from the results presented, though the full variable datasets consistently estimated SDMs as slightly more restrictive.

**TABLE 2:** The best model chosen using AICc for each species/group and the variables for modeling

*Plethodon*	Variables	Background	FC	RM	AUC_test_	AUC_diff_	OR_MTP_	OR_10_
*asupak*	bio12, bio13, bio16, bio19	jackknife	H	2	0.8686	0.0303	0.1667	0.3333
*elongatus*	bio1, bio2, bio6, bio12, bio14, bio15	checkerboard1	LQHP	3	0.9962	0.0007	0.0074	0.1061
*stormi*	bio1, bio2, bio3, bio4, bio12, bio14, bio15	jackknife	LQ	1	0.9991	0.0007	0.0159	0.1270
*elongatus‐stormi‐asupak*	bio1, bio2, bio6, bio14, bio15, bio17	checkerboard1	LQHP	3	0.9947	0.0004	0.0057	0.1046
*idahoensis*	bio4, bio7, bio8, bio9, bio15	jackknife	LQHPT	2	0.9678	0.0246	0.0089	0.1339
*larselli*	bio1, bio2, bio3, bio4, bio7, bio12, bio14, bio15	checkerboard1	LQH	3	0.9960	0.0018	0.0714	0.1265
*vandykei*	bio1, bio2, bio3, bio4, bio5, bio6, bio9, bio12, bio14, bio15	checkerboard1	LQHPT	3	0.9930	0.0014	0.0519	0.1408
*larselli‐vandykei*	bio1, bio2, bio3, bio4, bio5, bio6, bio12, bio14, bio15	checkerboard1	LQHPT	2	0.9893	0.0030	0.0377	0.1467
*larselli‐vandykei‐idahoensis*	bio1, bio2, bio3, bio4, bio5, bio6, bio9, bio10, bio14, bio15, bio17	checkerboard1	LQHPT	2	0.9803	0.0038	0.0032	0.1295
*dunni*	bio1, bio2, bio3, bio5, bio6, bio9, bio12, bio14, bio15	jackknife	LQH	2	0.9871	0.0051	0.0034	0.1153
*vehiculum*	bio1, bio2, bio3, bio4, bio7, bio15	jackknife	LQHPT	2	0.9804	0.0080	0.0015	0.1179
*dunni‐vehiculum*	bio1, bio2, bio3, bio4, bio5, bio7, bio10, bio11, bio15, bio18, bio19	jackknife	LQHPT	3	0.9789	0.0082	0.0011	0.1108

Note

RM = regularization multiplier (1, 2, or 3). FC = feature class (L, LQ, H, LQH, LQHP, LQHPT).

Niche breadth was small for all species, and SDM overlap was small to moderate (Table 3). As expected, there is less overlap between the current models and the future models from 2050 to 2070 and from the rcp4.5 to the rcp8.5 scenario, meaning that current models are more similar to year 2050 than 2070, and more similar to rcp4.5 than rcp8.5 (Table S3). Both the summed and averaged raster layers showed the same pattern (Table S4), where most species have an increase of suitable habitat available in the future projections (Figure 2). Similarly, the analysis counting grid cells with suitability scores above 0.6 and 0.8 suggests an expansion of suitable habitat from the future projections for most species (Tables 4 and S5). In general, the MIROC5 models were more restrictive than the CCSM4 models (Tables S4 and S5) and show more significant differences in the bioclimatic variables into the future (Table S6), indicating that MIROC5 predicts a bigger change in the environment for these salamanders in the PNW. As expected, there is a consistent significant increase in temperature for all species; however, rainfall appears to either stay the same (no significant difference) or increase. This increase is consistent particularly for bioclimatic variables 12 (Annual Precipitation), 13 (Precipitation of the Wettest Month), 16 (Precipitation of the Wettest Quarter), and 19 (Precipitation of the Coldest Quarter).

**TABLE 3:** Niche overlap for all species. Niche breadth is on the diagonal

Species	*asupak*	*elongatus*	*stormi*	*elongatus‐stormi‐asupak*	*idahoensis*	*larselli*	*vandykei*	*larselli‐vandykei*	*larselli‐vandykei‐idahoensis*	*dunni*	*vehiculum*	*dunni‐vehiculum*
*asupak*	0.6045	NA	NA	NA	NA	NA	NA	NA	NA	NA	NA	NA
*elongatus*	0.0955	0.0136	NA	NA	NA	NA	NA	NA	NA	NA	NA	NA
*stormi*	0.0456	0.0905	0.0062	NA	NA	NA	NA	NA	NA	NA	NA	NA
*elongatus‐stormi‐asupak*	0.1132	0.6808	0.2234	0.0199	NA	NA	NA	NA	NA	NA	NA	NA
*idahoensis*	0.1885	0.0019	0.0016	0.0053	0.0744	NA	NA	NA	NA	NA	NA	NA
*larselli*	0.1003	0.0276	0.0012	0.0263	0.0082	0.0094	NA	NA	NA	NA	NA	NA
*vandykei*	0.1354	0.0132	0.0028	0.0085	0.0069	0.1949	0.0240	NA	NA	NA	NA	NA
*larselli‐vandykei*	0.1669	0.0335	0.0028	0.0323	0.0072	0.4370	0.5937	0.0341	NA	NA	NA	NA
*larselli‐vandykei‐idahoensis*	0.2772	0.0204	0.0058	0.0207	0.2647	0.2737	0.4131	0.5212	0.0679	NA	NA	NA
*dunni*	0.1681	0.1556	0.0301	0.1924	0.0094	0.2100	0.2640	0.3934	0.3281	0.0382	NA	NA
*vehiculum*	0.2230	0.0791	0.0116	0.0963	0.0068	0.2254	0.4214	0.4923	0.4099	0.5579	0.0632	NA
*dunni‐vehiculum*	0.2232	0.1114	0.0123	0.1281	0.0091	0.2297	0.4168	0.4908	0.4300	0.6465	0.8674	0.0635

**TABLE 4:** Difference between the number of grid cells above 0.6 and 0.8 suitability for current versus future models for all species/groups

*Plethodon*	Model averaged by year	Difference 0.6	Difference 0.8
*elongatus*	2050	9,695	4,906
	2070	16,053	5,325
*stormi*	2050	−1736	−2012
	2070	−5382	−3692
*elongatus‐stormi‐asupak*	2050	27,105	18,698
	2070	16,185	14,242
*idahoensis*	2050	−23376	−34985
	2070	−24948	−37760
*larselli*	2050	82,092	59,220
	2070	137,432	90,194
*vandykei*	2050	−13978	−10645
	2070	−20199	−14223
*larselli‐vandykei*	2050	80,015	59,530
	2070	113,673	82,575
*larselli‐vandykei‐idahoensis*	2050	210,912	162,750
	2070	259,429	176,882
*dunni*	2050	33,141	28,242
	2070	57,561	37,761
*vehiculum*	2050	41,155	54,426
	2070	42,093	55,174
*dunni‐vehiculum*	2050	61,203	97,016
	2070	71,639	111,088

### 3.2 *Plethodon asupak*, *elongatus*, and *stormi*

*Plethodon elongatus* and *P. stormi* had little niche overlap in their SDMs (0.0905), and although both small, the niche breadth for *P. stormi* (0.0062) was half that *P. elongatus* (0.0136) (Table 3). This group showed the smallest combined range of any other group (Figure 2a), but was similar in size to *P. idahoensis*. The future SDM suggests a shift in habitat to the coast and out of the valley and a move north into the mountains. Overall, there appears to be more suitable habitat available for this group, except for *P. stormi* (Table 4). A similar pattern for *P*. *elongatus* alone is observed, and this species could potentially travel north along the coast (Figure S10). The *P. stormi* model shows a string of suitable habitat along the Cascade and Sierra Nevada Mountain Ranges, and this string is pronounced in the future model (Figure S11). Bioclimatic variable 15 (Precipitation Seasonality) consistently came out as being the most important to the models for all species in this group, followed by 12 (Annual Precipitation), 14 (Precipitation of the Driest Month), and 17 (Precipitation at the Driest Quarter) (Table S7).

### 3.3 *Plethodon idahoensis*, *larselli*, and *vandykei*

*Plethodon idahoensis* had little niche overlap with both *P. larselli* (0.0082) and *P. vandykei* (0.0069), while the niche overlap between the coastal species *P. larselli* and *P. vandykei* was much higher (0.1949) (Table 3). All three species had low niche breadth with *P. idahoensis* being the highest in this group and overall (0.0744), followed by *P. vandykei* (0.0240), then *P. larselli* (0.0094). Overall, in the *P. larselli‐vandykei* group, SDMs suggests more highly suitable habitat in the Cascades over the Coastal Mountains and expansion of suitable habitat in the North (Figure 2b); however, this is likely driven by *P. larselli,* as *P. vandykei* suitable habitat is expected to shrink (Table 4). For *P. larselli,* the future SDMs show an expansion in suitable habitat in all directions as far north as Vancouver and as far south as northern CA (Figure S12), while *P. vandykei* suitable habitat is severely restricted to small patches in the Cascade Mountains and Olympic Peninsula (Figure S13). The SDMs of *P. idahoensis* indicate a shift in suitable habitat to the northeast and encompasses a larger area (Figure 2c). Interestingly, even though *P. idahoensis* expands its range of suitable habitat, suitability scores are low, as demonstrated by an increase in summed suitability but a decrease in average suitability and the threshold grid cell counts. Additionally, the combined model for these three species shows an increase in suitable habitat around its entire distribution (Figure S14), but we do not consider this a suitable analysis as there is no overlap in distribution between *P. idahoensis* and *P. larselli* or *P. vandykei*. *Plethodon idahoensis* in general displays much less continuous suitable habitat and shows little niche overlap with the other two species. Bioclimatic variable 12 (Annual Precipitation) followed by 15 (Precipitation Seasonality) consistently came out as being the most important to the models for both *P*. *larselli* and *P. vandykei*, while several temperature variables were almost equally important for *P. idahoensis* (9: Mean Temperature of the Driest Quarter; 8: Mean Temperature of the Wettest Quarter; 4: Temperature Seasonality; 7: Temperature Annual Range) (Table S7).

### 3.4 *Plethodon dunni* and *vehiculum*

*Plethodon dunni* and *P. vehiculum* had the highest niche overlap among any of the pairwise comparisons (0.5579), and also the most overlap with other species (Table 3). *Plethodon dunni* shared niche space with *P. larselli* (0.2100) and *P. vandykei* (0.2640), similar to *P. vehiculum* sharing niche space with *P. larselli* (0.2254) and *P. vandykei* (0.4923). Of all the species analyzed, *P. vehiculum* had the second highest niche breadth (0.0632), followed by *P. dunni* (0.0382). The *P. dunni‐vehiculum* group future SDM suggests an increase in habitat suitability in most of its current range, except the southern portion of its range and in the valley, and far more suitable habitat in the north (Figure 2d). Expansion of suitable habitat is clear for this group and both species individually (Table 4). In *P. dunni,* the southwestern portion of the SDMs is greatly reduced along the Pacific coast but expands largely in the north and in the Cascades (Figure S15). The coastal region from mid‐OR to around the Olympic Peninsula for *P. vehiculum* has the most notable increase in climate suitability (Figure S16). These results coincide with model projections for the mid‐Holocene, when the climate was much warmer, in that suitable habitat for these species was predicted to be larger and more north and coastal (Pelletier & Carstens, 2016). Bioclimatic variables 7 (Temperature Annual Range) and 15 (Precipitation Seasonality) were the most important for *P. vehiculum*, while variables 12 (Annual Precipitation) and 14 (Precipitation of the Driest Month) were the most important for *P. dunni*, and 7 (Temperature Annual Range) and 19 (Precipitation of Coldest Quarter) were the most important for their group model (Table S7).

## 4 DISCUSSION

Protecting biodiversity is a widely accepted concept that results in benefits for humans on many levels (Burch‐Brown & Archer, 2017), and the PNW is often considered an area of conservation prioritization (Brooks et al., 2006). Protections may happen at many levels from populations to communities. Understanding how species will respond to changes in climate will aid planning strategies moving forward, especially given that species responses are not homogeneous (Rapacciuolo et al., 2014). Furthermore, focusing on species groups that form a clade and share geographic space can consolidate conservation efforts. In this case, we can consider looking at a group of four responses to climate change, rather than eight separate responses for each species. For example, the *P. dunni‐vehiculum* analysis is likely enough to consider for protecting this lineage. This body of work contributes to a better understanding of a small group of salamanders, which is especially important given recent declines in amphibian populations (Stuart et al., 2004) and limited studies on salamanders that project distributions under future climate scenarios (Zellmer et al., 2020).

The species current estimated niche matched those of their current distributions, indicating that the climate variables available for use are capturing the environmental niche of these species (Baselga et al., 2012). It is not unexpected that these 19 bioclimatic layers provide a reasonable niche estimate, given that amphibians are constrained by precipitation and temperature (Buckley & Jetz, 2007). Most species in this group will not experience a decrease in suitable habitat, and in many cases, it expands. Most expand northward, with the exception of the most southern group (*elongatus‐stormi‐asupak*). This group seems to be at the highest risk for a contraction in their range and shifts toward the coast. This is similar to results from eastern *Plethodon* salamanders where southern species with small ranges displayed higher habitat loss, particularly those in drier ecoregions (Jacobsen et al., 2020; Milanovich et al., 2010; Sutton et al., 2015). *Plethodon stormi* in particular shows a pattern of expansion along the Cascade and Sierra Nevada Mountains indicating that this species may be especially restricted to the mountains and is the only species that might move southward. Following the mountain ranges in this geographic area is not an uncommon pattern (Matocq, 2002; Moritz et al., 1992), and given that several species in this group are mainly found in wet rocky talus slopes (Herrington & Larsen, 1985; McIntyre et al., 2006; Suzuki et al., 2008), it would not be surprising if salamanders in the PNW become more restricted to higher elevations. This might hinder their movement through valleys; however, there are many instances where many of these species have been found in what is not considered their preferred habitat (Corkran & Thoms, 2006).

Even though climate predictions suggest an increase in temperature in the PNW, we suspect we are not seeing an overall decrease in suitable habitat for most of these species because they are more dependent on precipitation and can spend the hot dry months underground, as is currently demonstrated by the southern species in this group (Bury & Pearl, 1999). This is not surprising due to amphibians’ strong association with moist environments (Buckley & Jetz, 2007), particularly terrestrial salamanders (Peterman & Semlitsch, 2014). Though change in average temperature is often a central focus of climate change research, other variables can be important factors in a species’ ecological niche. These may not be the same for species in similar geographic areas and might be contradictory with the expectations of a warming climate, but the exposure to temperature increases in these species could be minimal (Gade et al., 2020; Rapacciuolo et al., 2014). The extended growing season and potential for an increase in precipitation will likely keep canopy cover high retaining moisture and limiting solar radiation and vapor pressure, which could be beneficial for these salamanders. This is further supported by a study done on a group of eastern Plethodontid salamanders that suggests surface activity may increase under future climate conditions (Gade et al., 2020). Furthermore, these species breed terrestrially, allowing them freedom from bodies of water, and therefore limiting their dependency on the same environmental cues that would be necessary for pond or stream breeding.

Because Plethodontids spend most of their time underground, the ability to assess dispersal in this group is difficult, but it is thought to be limited (Ovaska, 1988; Smith & Green, 2005). Due to their risk of desiccation, open areas are often considered difficult for surface activity in these salamanders; however, studies of their migration in deforested areas as compared to their forested counterparts demonstrated no such limitation (Marsh et al., 2004). Additionally, studies exploring both past and contemporary patterns in range expansion suggest salamanders can disperse very long distances, though it varies among individuals and species (Fonte et al., 2019; Lowe, 2010; Pelletier & Carstens, 2016). We suspect that dispersal might be a strong predictor in how these species respond to climate change and that dispersal is not necessarily tied to habitat suitability because landscape features in relation to the resistance of gene flow have been found to vary across geographic areas within a single species of an eastern *Plethodon* salamander (Burgess & Garrick, 2020).

Alternatively, metapopulations arise within species as a result of limited isolation, a common pattern in salamanders (Smith & Green, 2005), and open areas within forests are a continuing result of deforestation. Continued loss of habitat may result in fragmentation of populations, possibly resulting in species divergence, rather than extinction (Jackson & Sax, 2010). The species within this group display either a single small distribution, several disjunct distributions, or large distributions with population genetic structure. Similar to its eastern counterparts, western *Plethodon* are highly associated with cool moist montane climates and because of this niche conservatism across species, those that are geographically isolated diverge, a pattern seen many times in eastern *Plethodon* (Kozak & Wiens, 2006). In fact, there are well over five times the number of eastern *Plethodon* salamanders than western *Plethodon* and it is possible that the western *Plethodon* salamanders may follow a similar pattern if they become more restricted to higher elevations. *Plethodon* salamanders have a long history in the United States and have seen increased diversification during warmer time periods (Vieites et al., 2007).

## 5 CLIMATE MODEL LIMITATIONS

Climate modeling has several limitations based on assumptions that are made regarding the data used and the methods used to assess potential changes in the key factors of a species’ survival (Milanovich et al., 2010). While some methods might outperform MaxEnt in some scenarios (Vasilakos et al., 2020), and could be explored with other data layers, several studies on salamanders have used similar methods successfully in recent years and offer relevant comparisons to our study (Antunes et al., 2021; Zellmer et al., 2020). Yet, such modeling lacks quantification of a species’ ability to disperse, ignores potential for evolutionary change, and fails to account for biotic impacts, assuming that climate is the greatest driving factor of a species distribution (Jackson & Sax, 2010; Saupe et al., 2012; Soberón et al., 2007). Despite these limitations, such modeling efforts are beneficial in order to determine the validity of various predictions in the future (Esser et al., 2019; Zhang et al., 2019). Finally, in order to computationally assess a species’ niche with fidelity, many of the aforementioned factors could be used together. To achieve these feats would be a tremendous undertaking, as consideration of the implications of a high number of variables and their interactions will compound the need for more species‐specific data and may result in over‐parameterizing models. Therefore, distribution modeling is worthwhile as a stepping stone to achieve greater understanding of the impact individual environmental factors have on species’ distributions.

## 6 CONCLUSIONS

Even though the distribution of these salamanders does not appear to be greatly affected by climate change in the near future, species with small distributions are usually at high risk for extinction, and SDM predictions become less reliable for species with smaller distributions (Schwartz et al., 2006); therefore, it is important that *P. vandykei*, *P. stormi,* and *P. asupak* be closely monitored. Factors that may influence *Plethodon* salamanders are those pertaining to moisture and solar radiation. Given the importance of moisture for a species that spends significant amounts of time and breeds underground, soil composition, salinity, pH, nutrient composition, and moisture retention could be essential for the habitat of *Plethodon* salamanders. Though some studies have considered soil type and rock coverage in assessing species distributions (Suzuki et al., 2008), none have studied a correlation between climatic variables such as precipitation with soil composition and retention, and how that may impact subterrestrial species response to climate change. This will be especially important for species with small ranges that are currently at risk on some level (e.g., *P. stormi*, and *P. larselli*; Table 1). Finally, we downloaded spatial layers of protected areas (UNEP‐WCMC & IUCN, 2020) and found that a total of 551 (32%) of our GPS coordinates fell within some sort of protected area (Table 1) and recommend a concerted effort in maintaining currently protected areas in the PNW.

## CONFLICT OF INTEREST

The authors declare no conflicts of interest.

## AUTHOR CONTRIBUTION

**Sir Nottingham:** Conceptualization (supporting); Data curation (lead); Formal analysis (lead); Writing‐original draft (equal); Writing‐review & editing (equal). **Tara A. Pelletier:** Conceptualization (lead); Data curation (supporting); Formal analysis (supporting); Funding acquisition (lead); Writing‐original draft (equal); Writing‐review & editing (equal).

## Supporting information

Fig S1‐S25

Table S1‐S8

## Data Availability

All data and scripts are available on GitHub: https://github.com/shastara/plethodon_niche
