# Supplementary material for: The impact of climate change on western Plethodon salamanders’ distribution
Source: Ecol Evol. 2021 Jun 29;11(14):9370–84. doi: 10.1002/ece3.7735 (PMC8293714; doi:10.1002/ece3.7735)
Supplement: Supplementary file 1 — Fig S1‐S25 [file ECE3-11-9370-s001.pdf]

Supplemental figures for:

The impact of climate change on western *Plethodon* salamanders' distribution

Sir Nottingham and Tara A Pelletier*
Department of Biology, Radford University, Radford, Virginia, 24142
*Corresponding Author: tpelletier@radford.edu

List of supplemental figures

S1: GPS coordinates for *asupak*
S2: GPS coordinates for *dunni*
S3: GPS coordinates for *elongatus*
S4: GPS coordinates for *larselli*
S5: GPS coordinates for *idahoensis*
S6: GPS coordinates for *stormi*
S7: GPS coordinates for *vandykei*
S8: GPS coordinates for *vehiculum*
S9: current and future species distribution model for *asupak*
S10: current and future species distribution model for *elongatus*
S11: current and future species distribution model for *stormi*
S12: current and future species distribution model for *larselli*
S13: current and future species distribution model for *vandykei*
S14: current and future species distribution model for *larselli*-*vandykei*-*idahoensis*
S15: current and future species distribution model for *dunni*
S16: current and future species distribution model for *vehiculum*
S17: all variable current species distribution model for *asupak*
S18: all variable current species distribution model for *elongatus*
S19: all variable current species distribution model for *stormi*
S20: all variable current species distribution model for *idahoensis*
S21: all variable current species distribution model for *larselli*
S22: all variable current species distribution model for *vandykei*
S23: all variable current species distribution model for *dunni*
S24: all variable current species distribution model for *vehiculum*
S25: Bayesian Phylogenetic tree based on cytochrome b

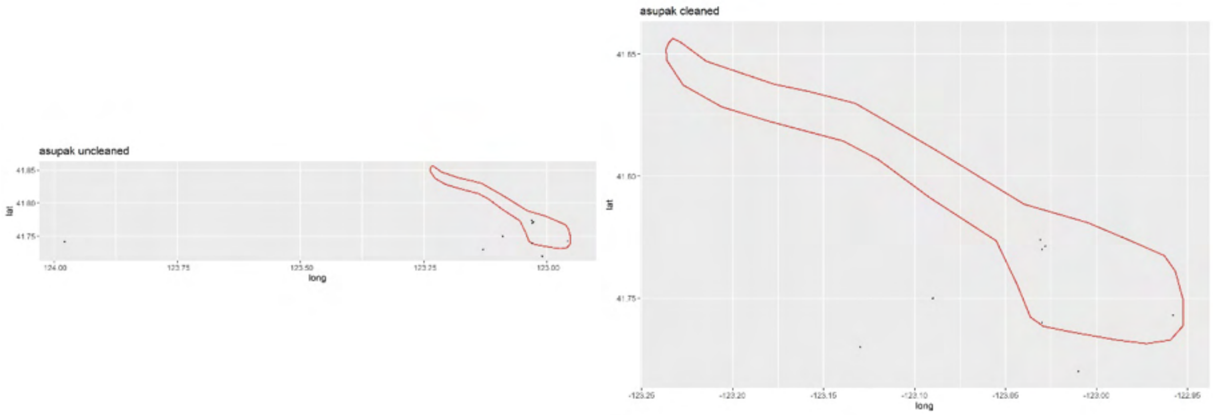

Figure S1. ***Plethodon asupak.*** A single outlier point to the west was removed due to its’ distance from established range.

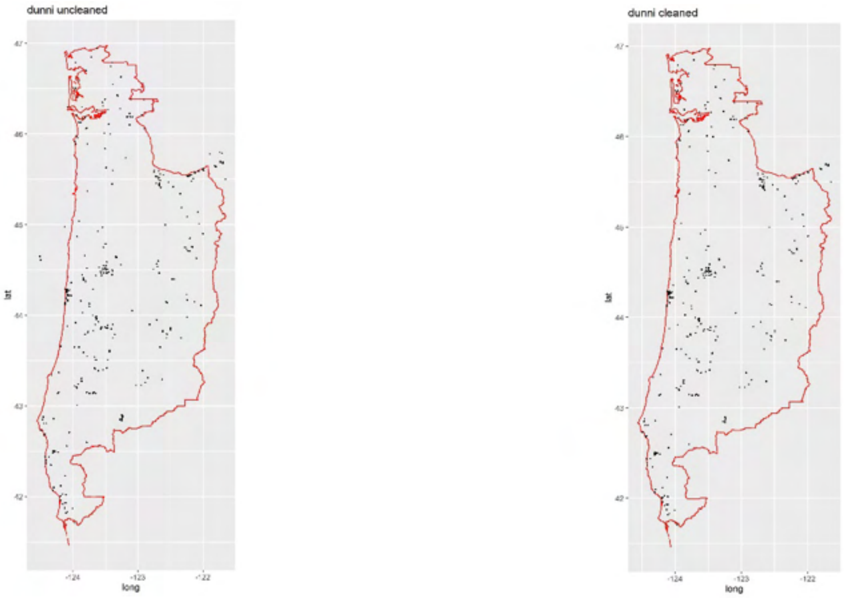

Figure S2. ***Plethodon dunni.*** All points west of the IUCN distribution were removed because these points fall in the ocean. On the eastern side of the established range, points that fell north of the Columbia River were removed as they are likely misidentified because they are not known to be found north of the Columbia River east of the valley.

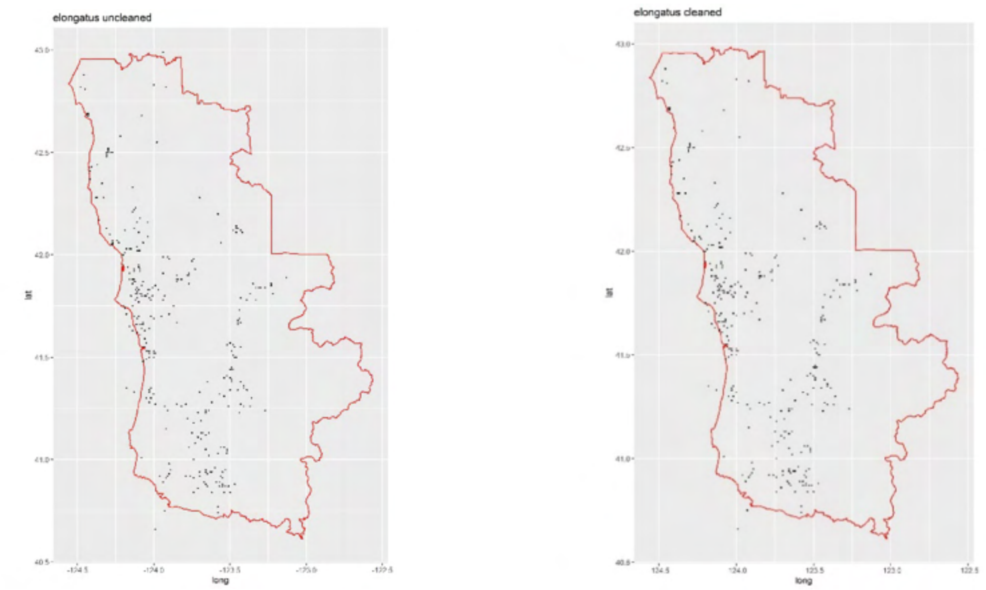

Figure S3. ***Plethodon elongatus.*** A single point was removed from outside of the northern area because the possibility of being a mis-identified *P. vehiculum* is high.

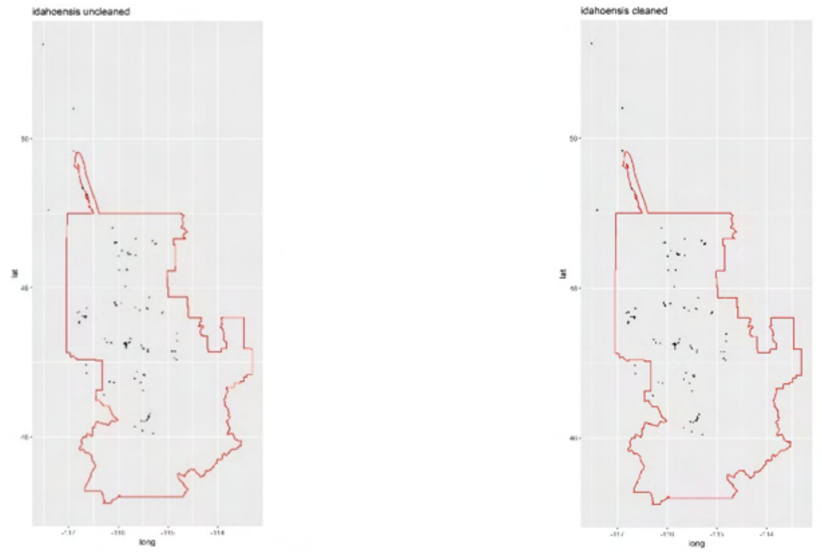

Figure S4. ***Plethodon idahoensis.*** No points were removed due to idahoensis' isolation from other *Plethodon* species.

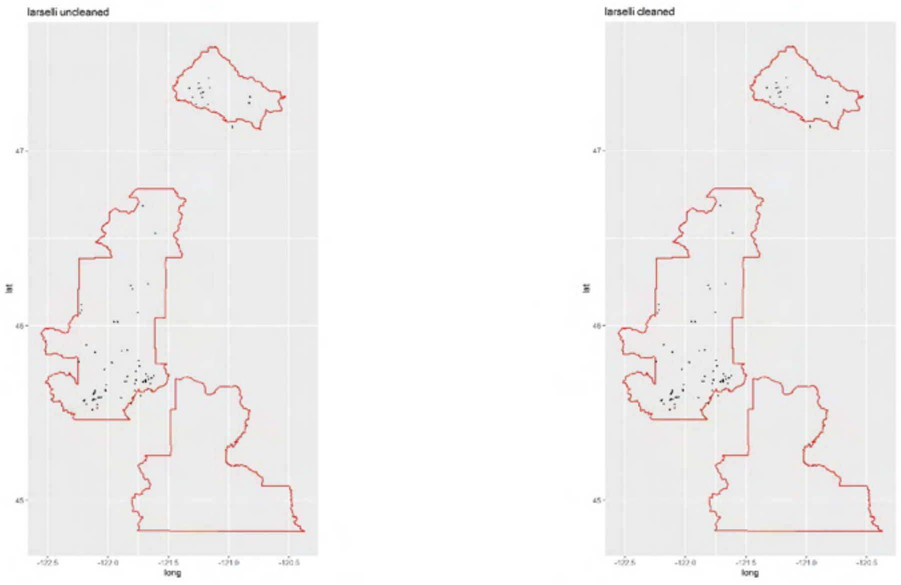

Figure S5. ***Plethodon larselli.*** No points were removed from this dataset.

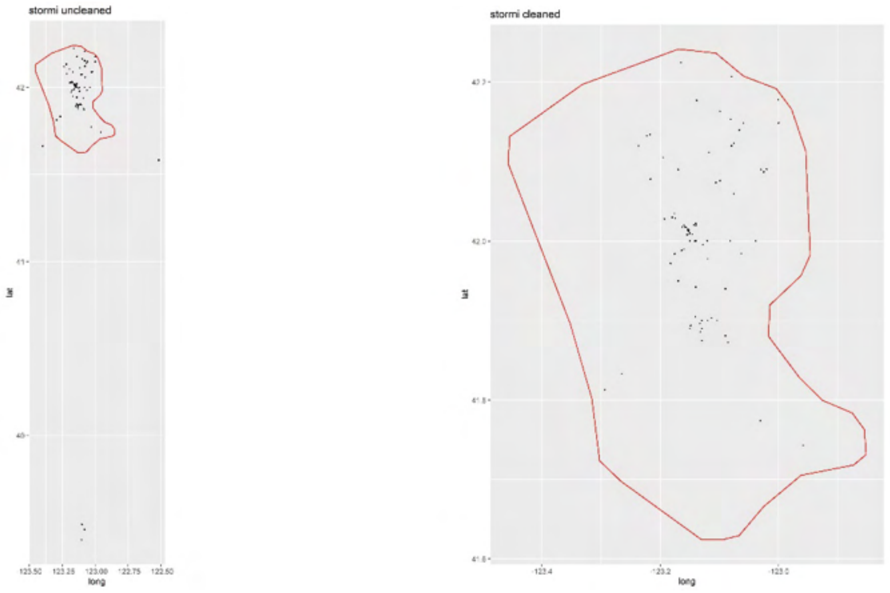

Figure S6. ***Plethodon stormi.*** Outlier points found far south of the established region were removed as well as less distant points to the east and west of the IUCN region because of the likely occurrence of a *P. elongatus* mis-identification.

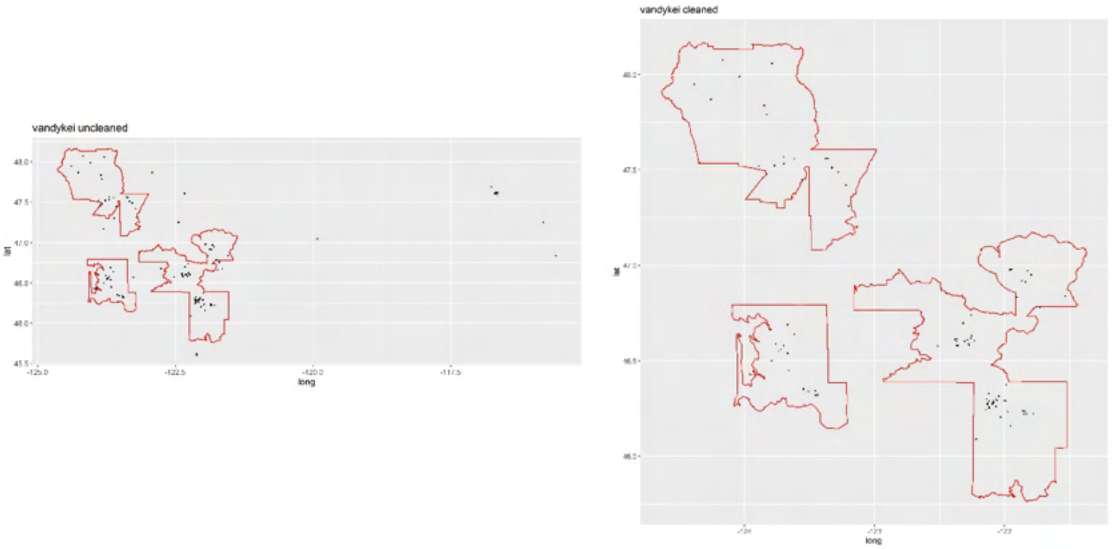

Figure S7. *Plethodon vandykei.* All points outside of the established IUCN regions were removed because the possibility of being a mis-identified *P. vehiculum* is high.

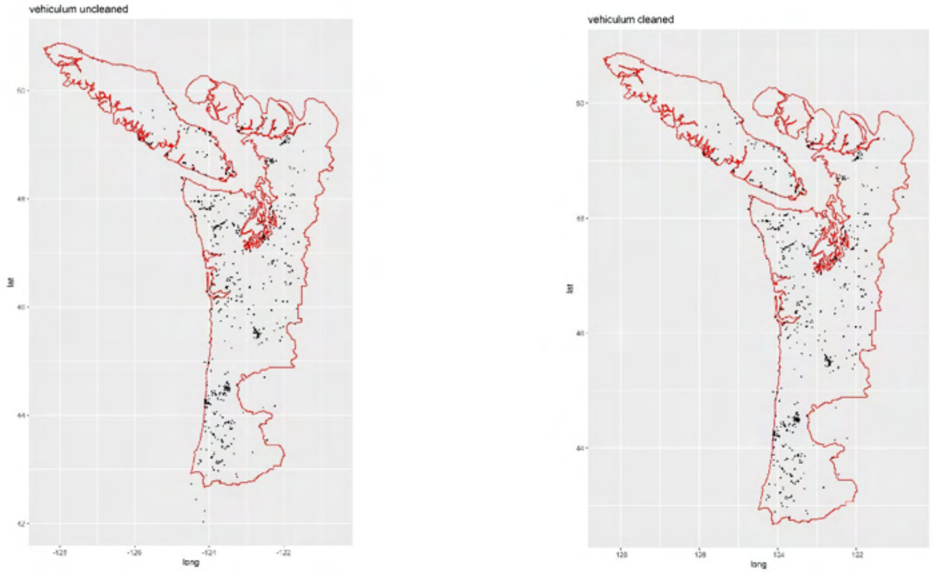

Figure S8. *Plethodon vehiculum.* Only points found south of the IUCN region were removed because of the likely occurrence of a *P. elongatus* mis-identification.

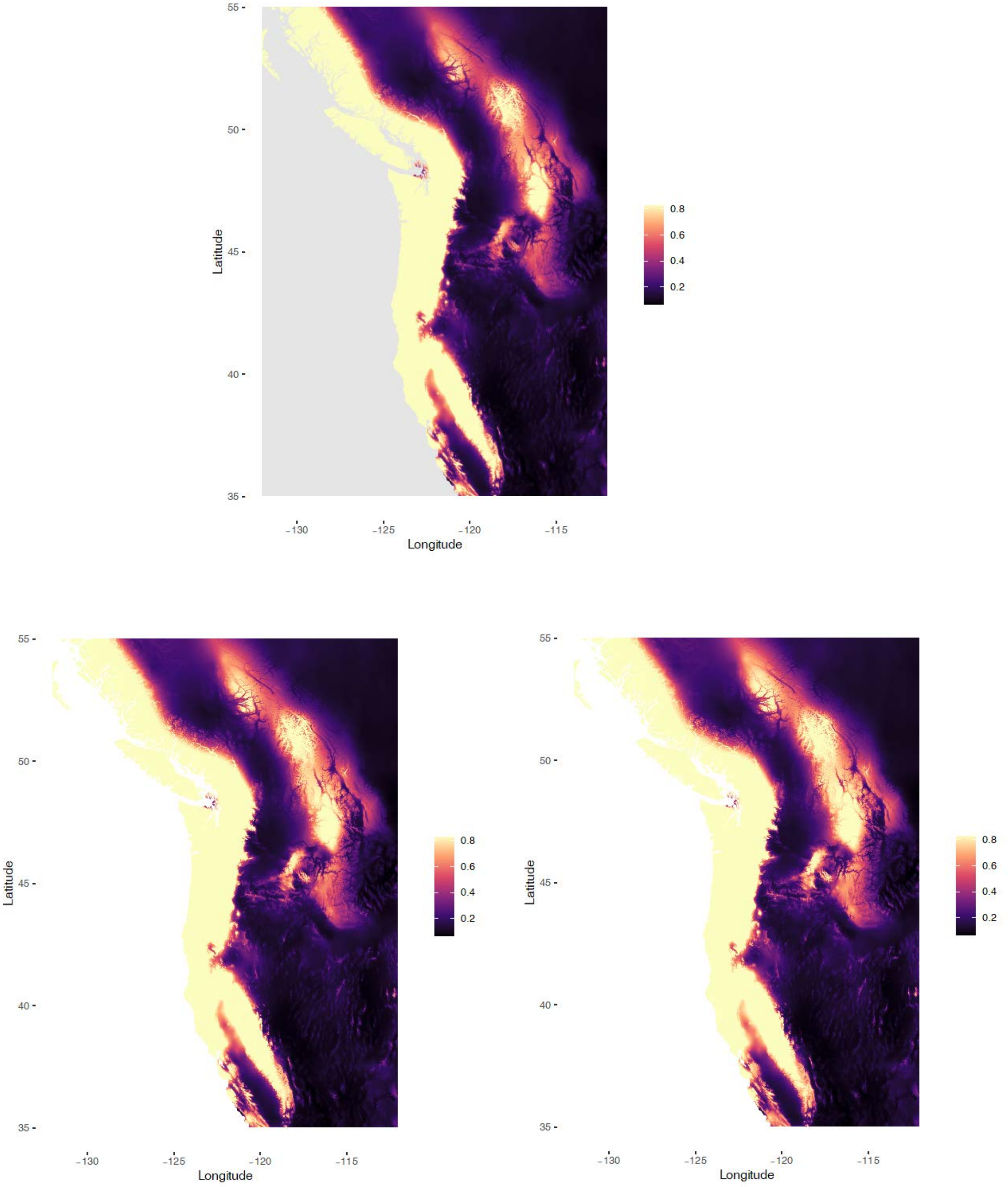

2050 **2070**

Figure S9: ***Plethodon asupak*** current and future species distribution model.

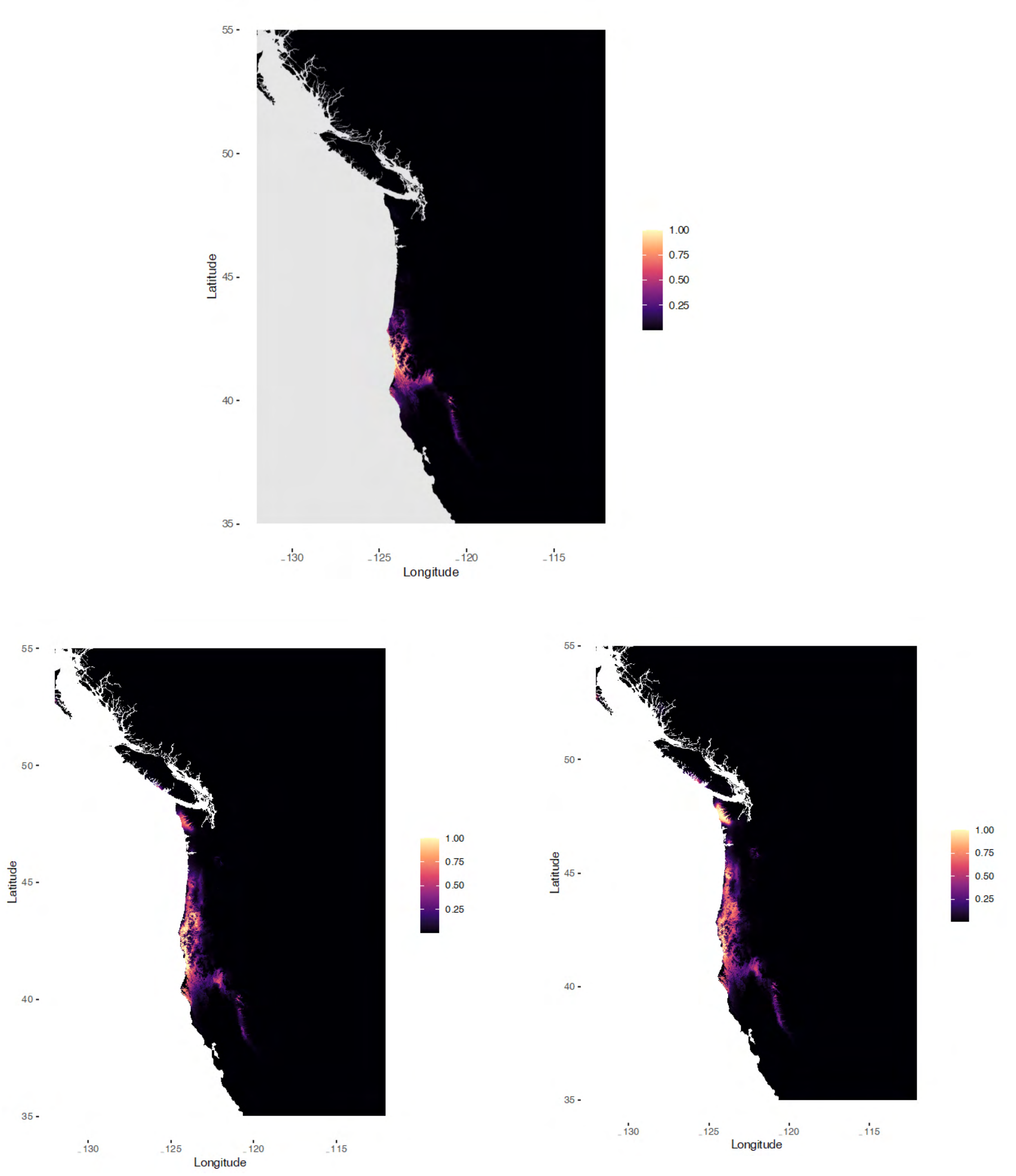

2050 **2070**

Figure S10: ***Plethodon elongatus*** current and future species distribution model.

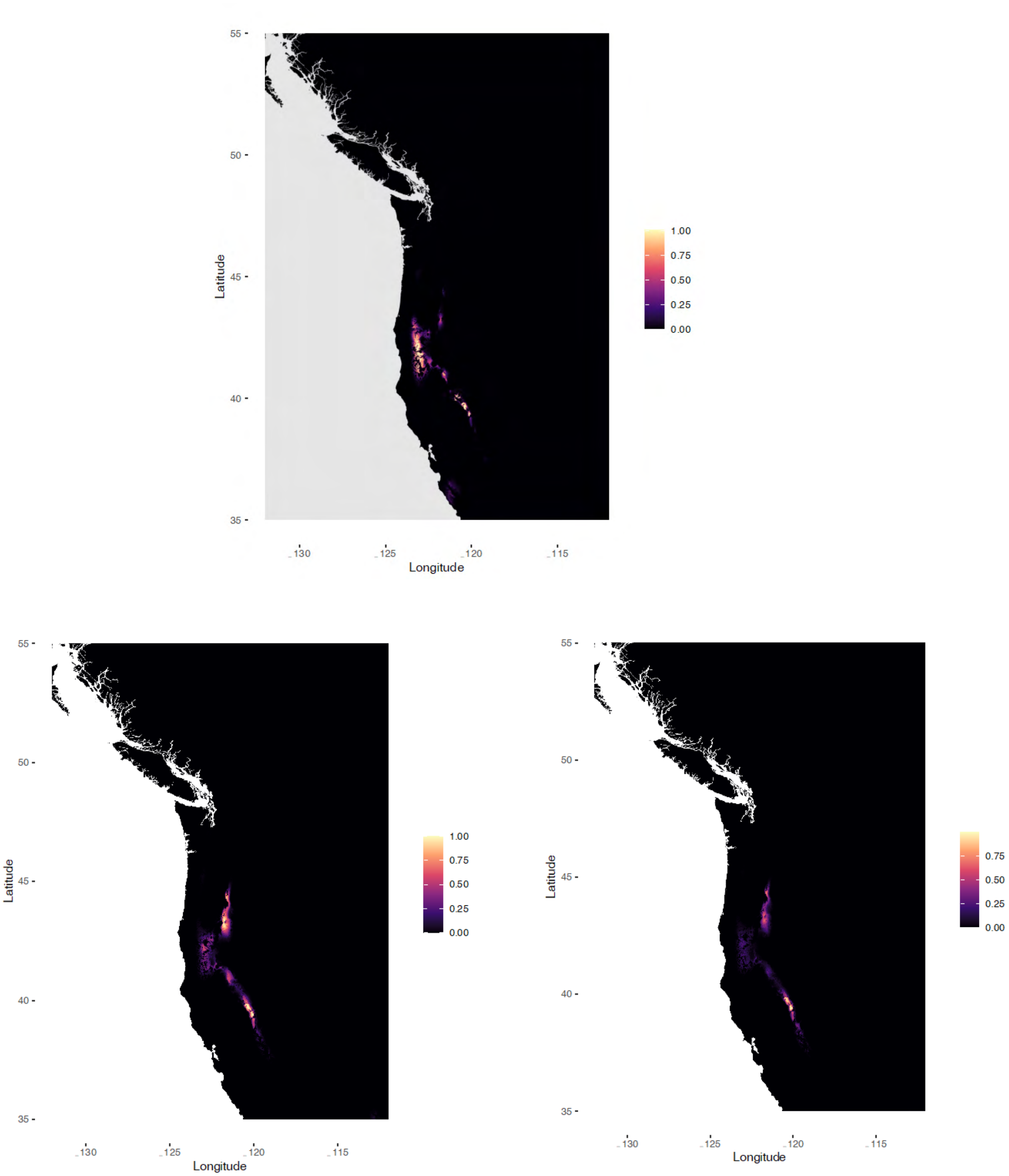

2050 **2070**

Figure S11: ***Plethodon stormi*** current and future species distribution model.

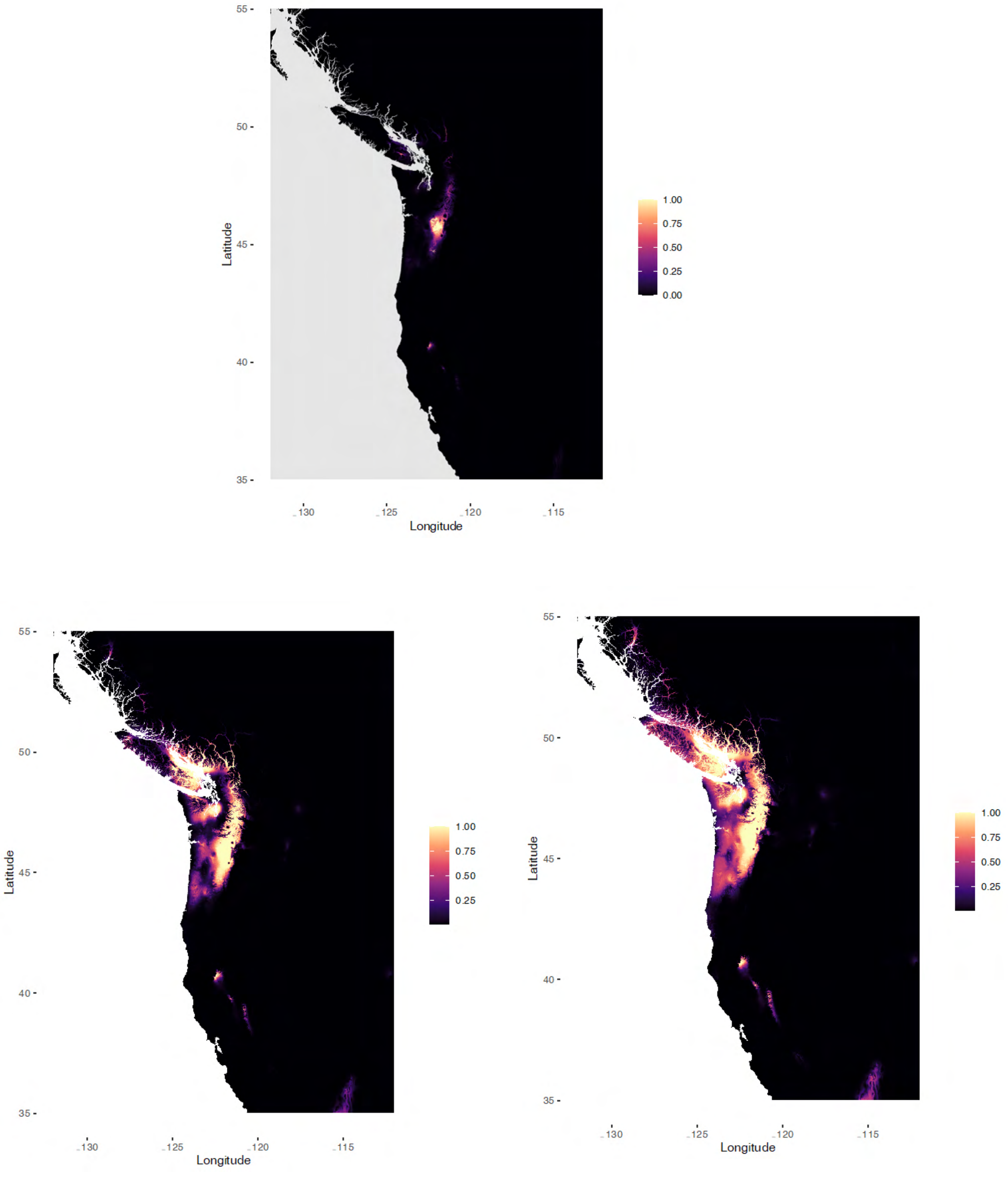

2050

2070

Figure S12: ***Plethodon larselli*** current and future species distribution model.

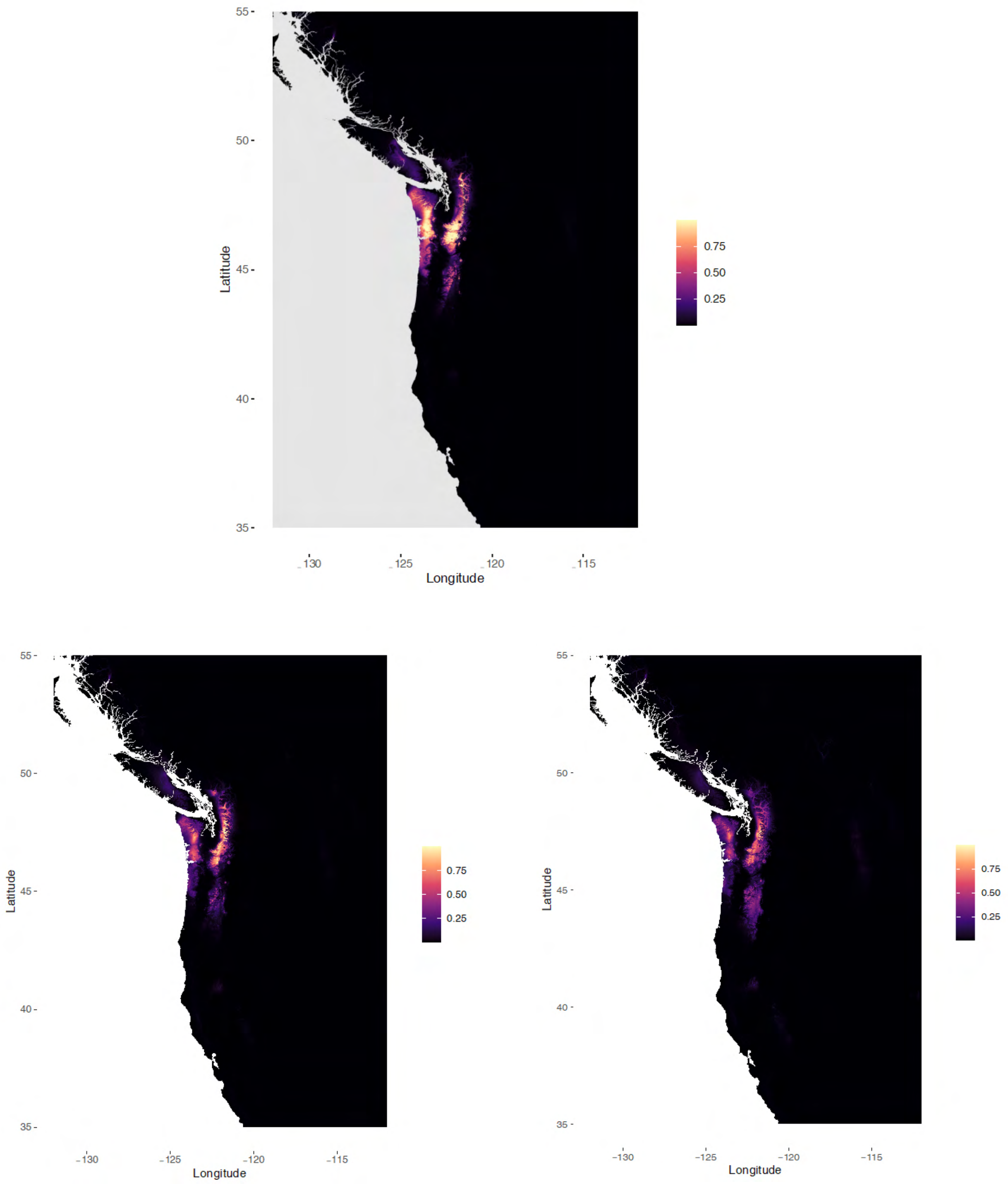

2050 **2070**

Figure S13: ***Plethodon vandykei*** current and future species distribution model.

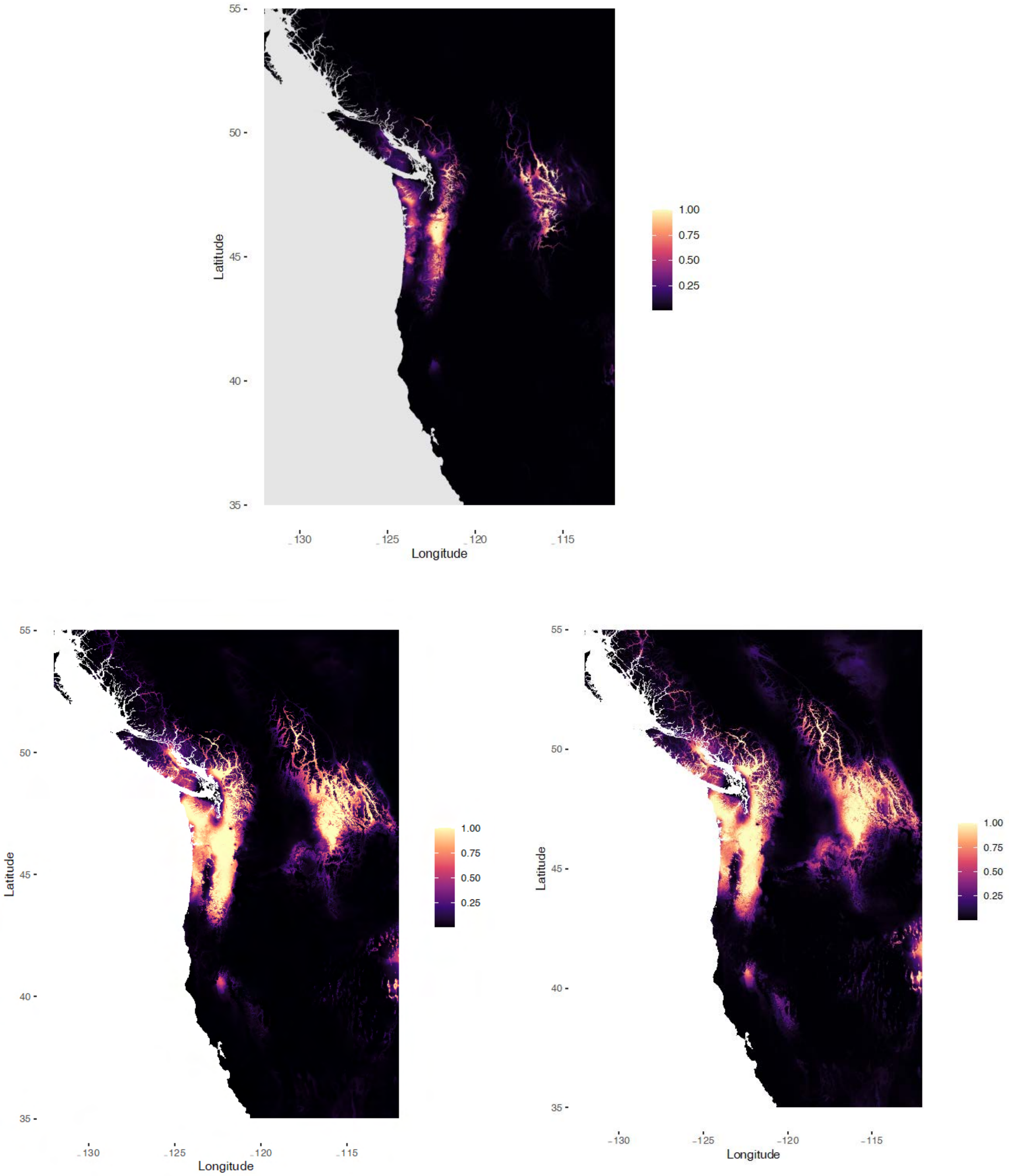

2050 **2070**

Figure S14: ***Plethodon larselli-vandykei-idahoensis*** current and future species distribution model.

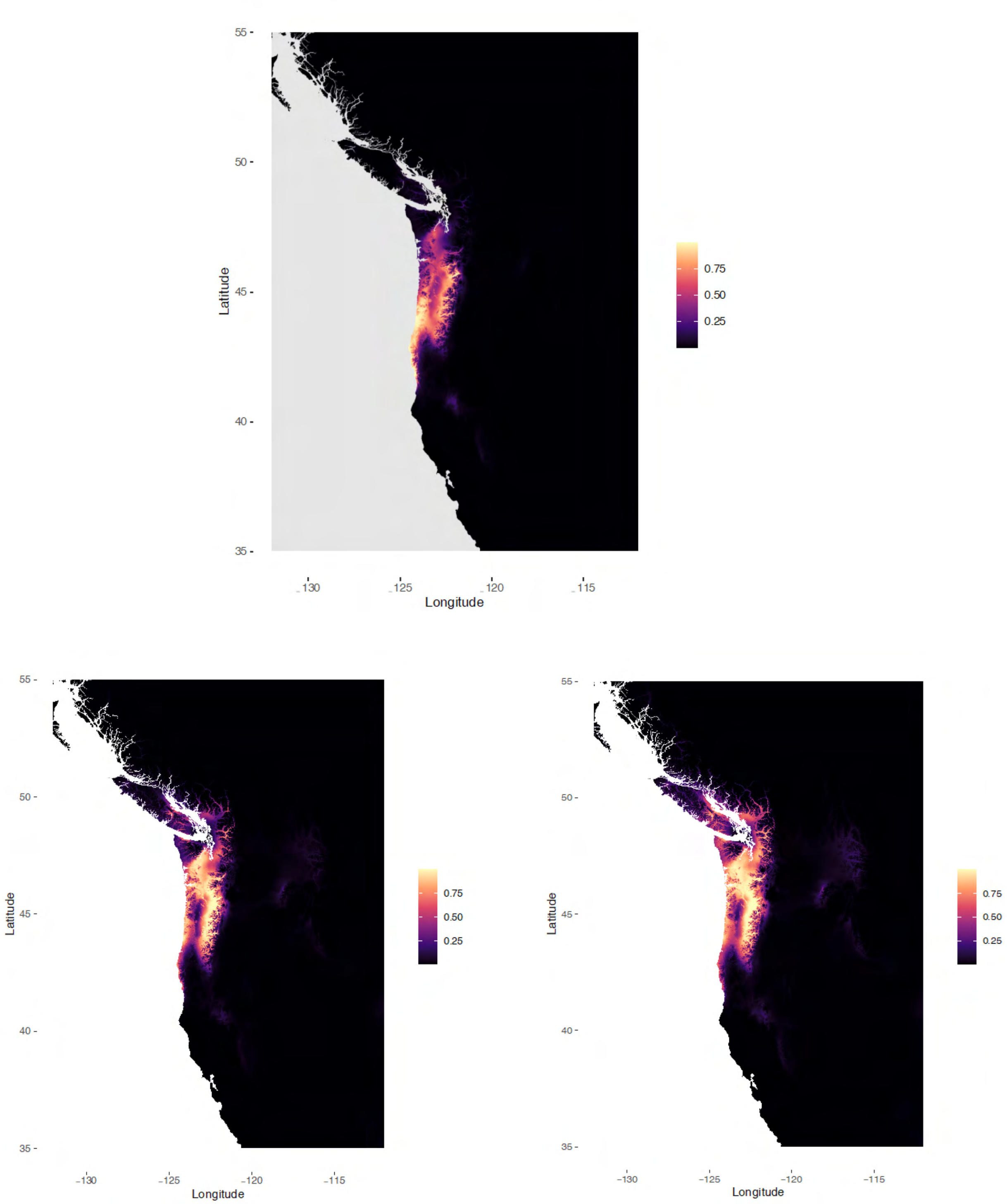

2050 **2070**

Figure S15: ***Plethodon dunni*** current and future species distribution model.

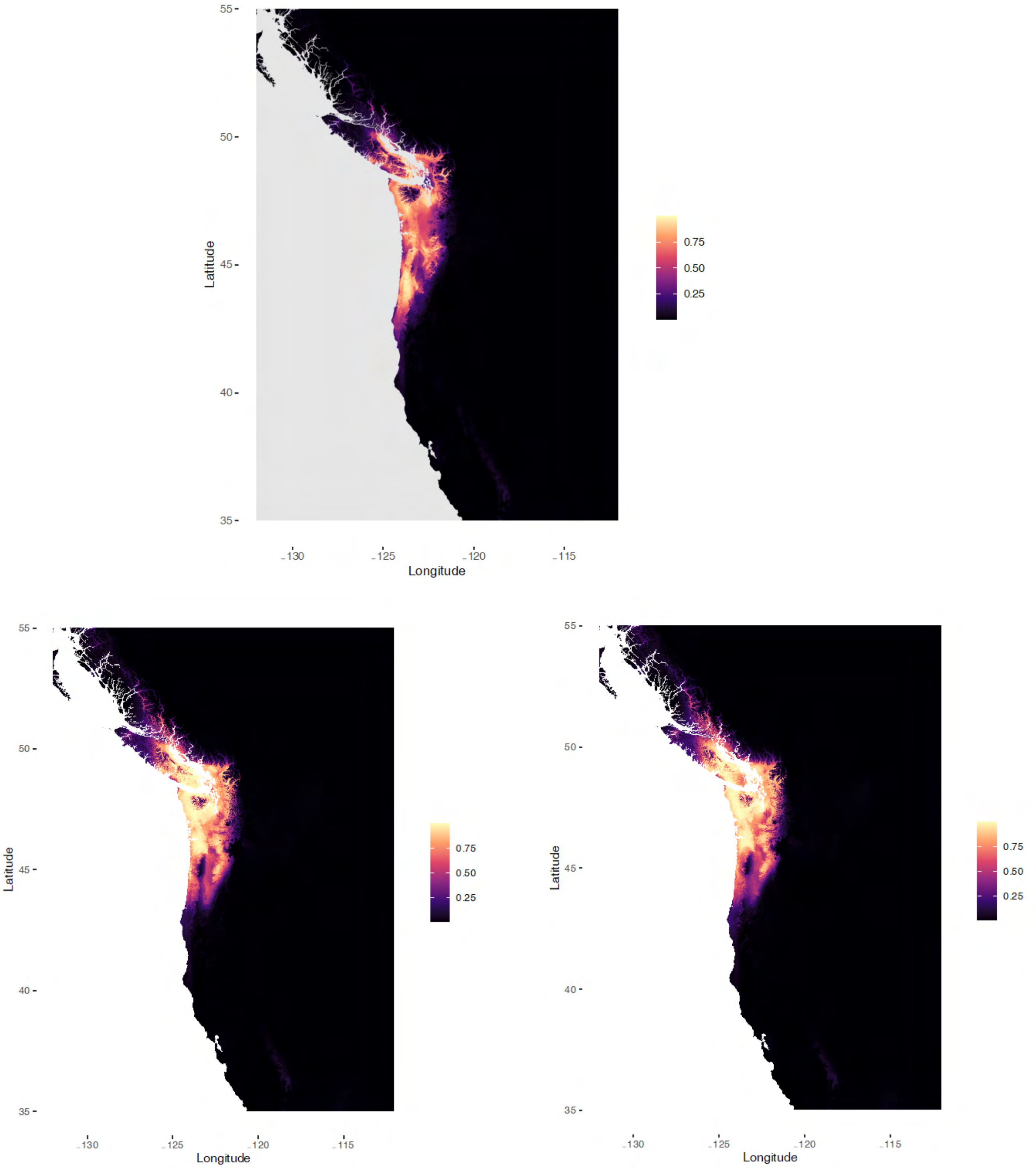

2050 **2070**

Figure S16: ***Plethodon vehiculum*** current and future species distribution model.

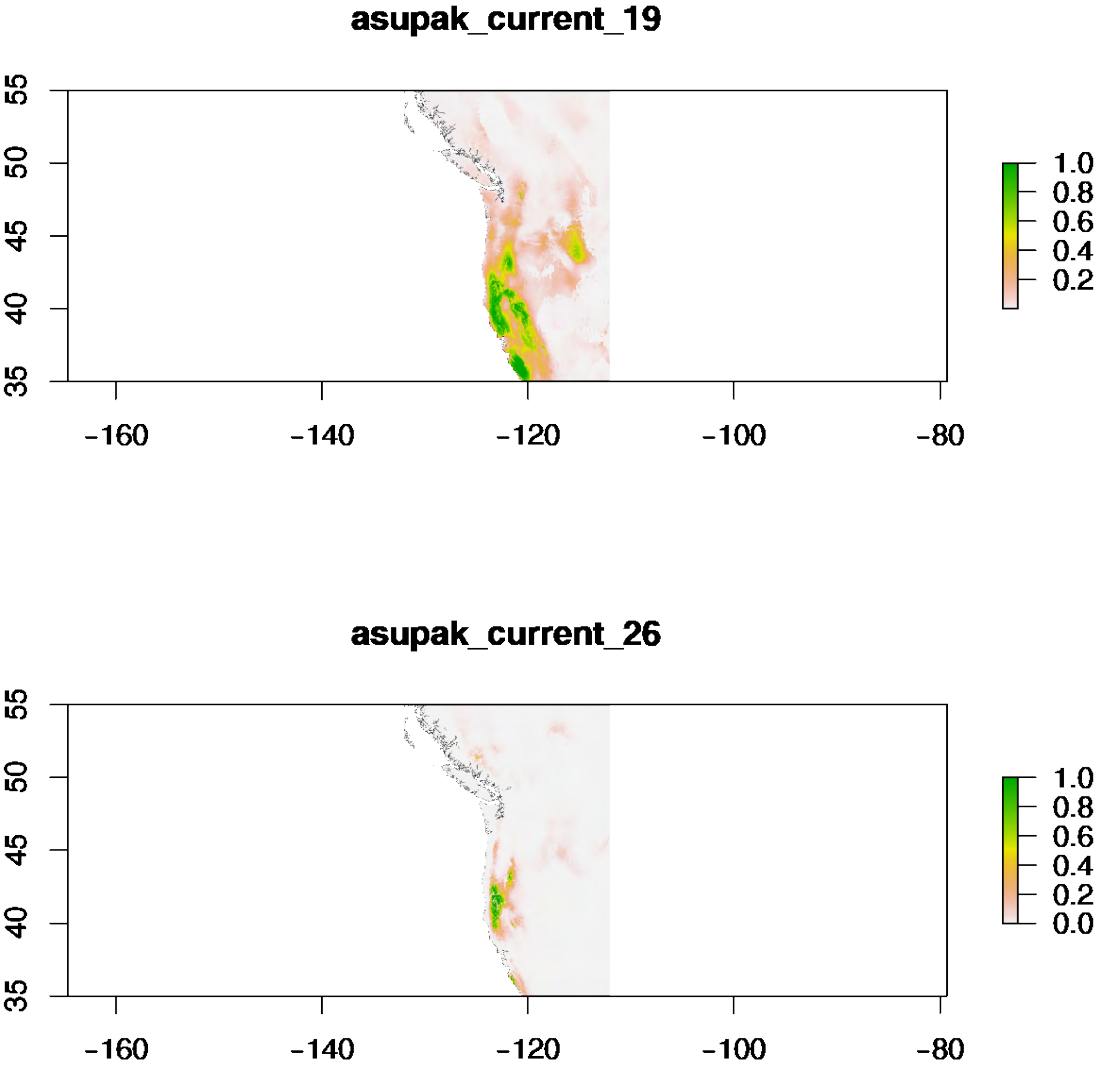

Figure S17: ***Plethodon asupak*** current species distribution model before removal of correlated variables (all 19 bioclimatic (AUC= 0.971) variables and including elevation, solar radiation, wind speed, and water vapor pressure totaling 26 (AUC= 0.998)).

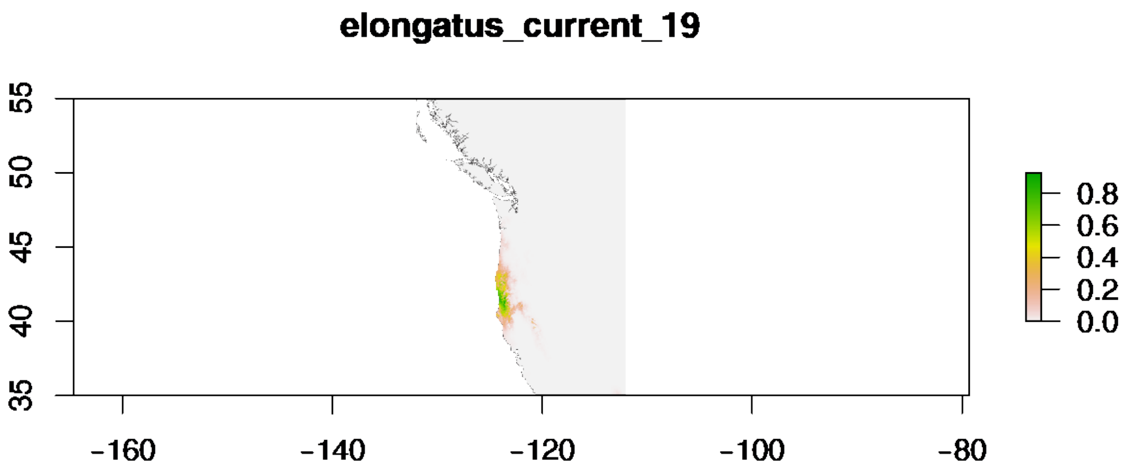

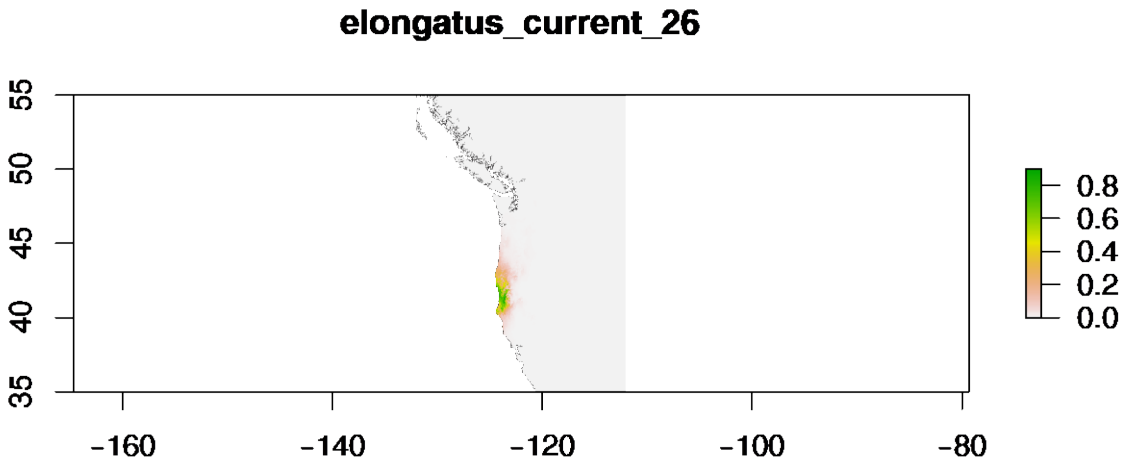

Figure S18: ***Plethodon elongatus*** current species distribution model before removal of correlated variables (all 19 bioclimatic (AUC= 0.987) variables and including elevation, solar radiation, wind speed, and water vapor pressure totaling 26(AUC= 0.988)).

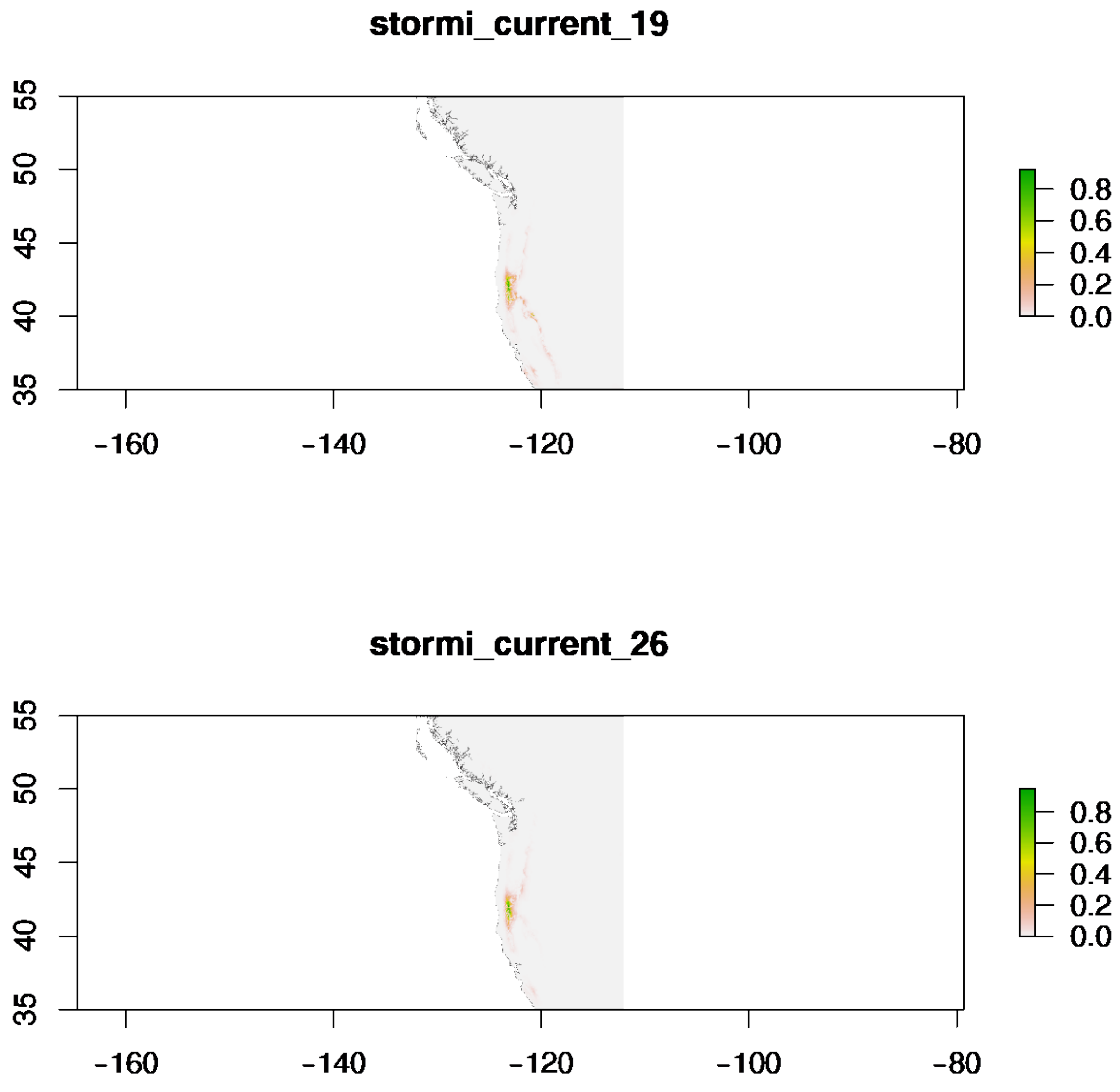

Figure S19: ***Plethodon stormi*** current species distribution model before removal of correlated variables (all 19 bioclimatic (AUC= 0.997) variables and including elevation, solar radiation, wind speed, and water vapor pressure totaling 26 (AUC= 0.998)).

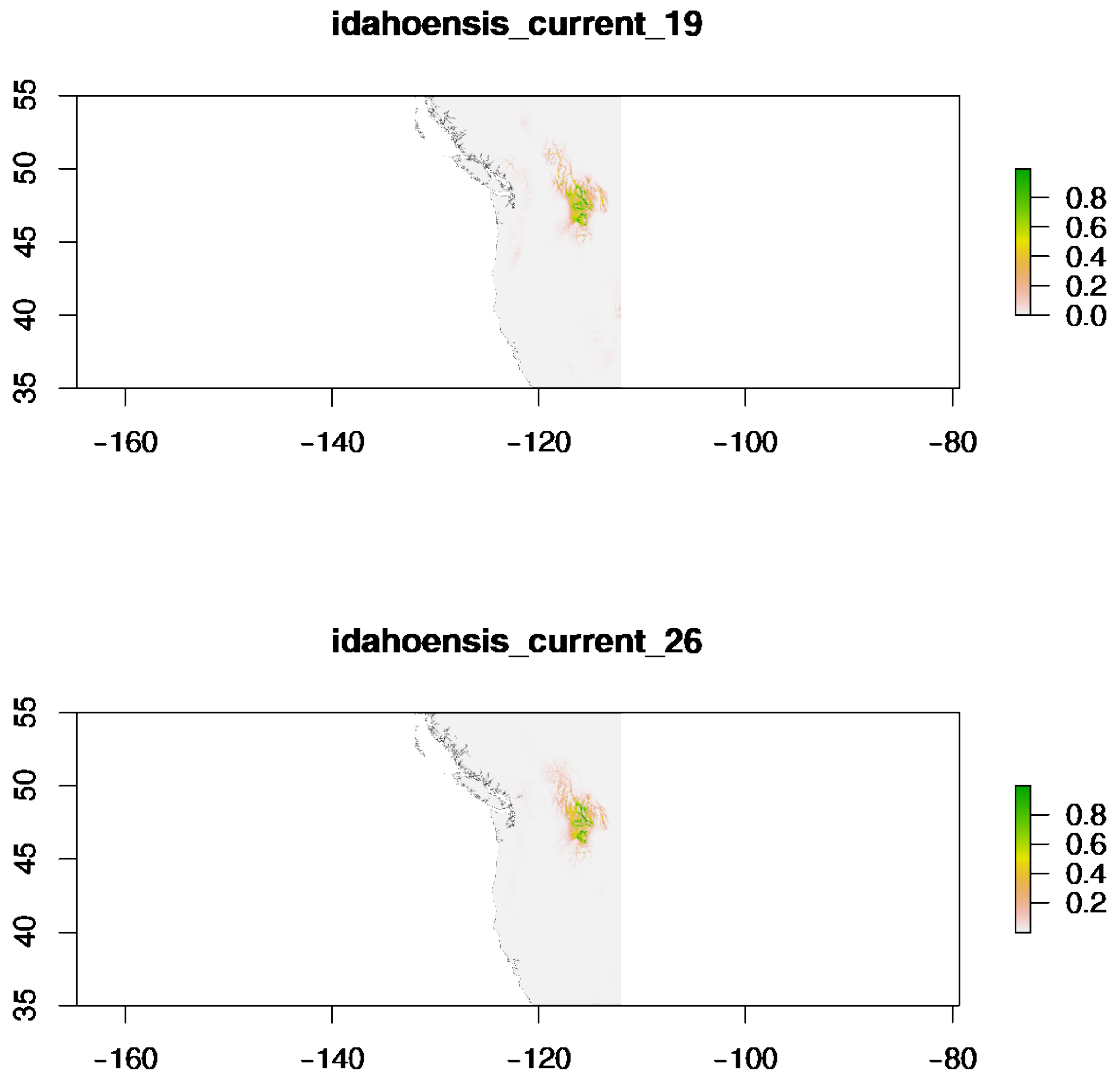

Figure S20: ***Plethodon idahoensis*** current species distribution model before removal of correlated variables (all 19 bioclimatic (AUC= 0.990) variables and including elevation, solar radiation, wind speed, and water vapor pressure totaling 26 (AUC= 0.991)).

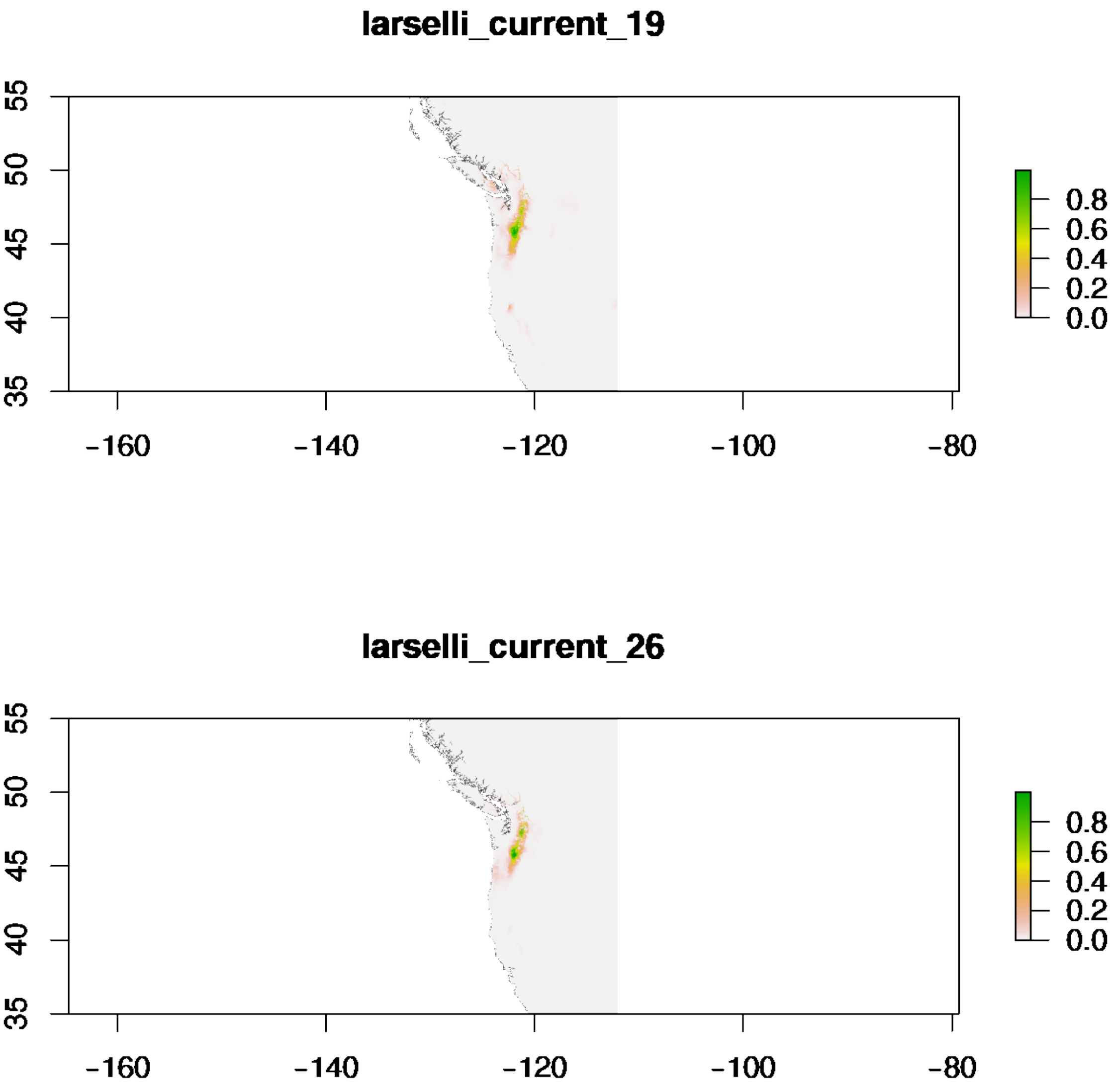

Figure S21: ***Plethodon larselli*** current species distribution model before removal of correlated variables (all 19 bioclimatic (AUC= 0.995) variables and including elevation, solar radiation, wind speed, and water vapor pressure totaling 26 (AUC= 0.9996)).

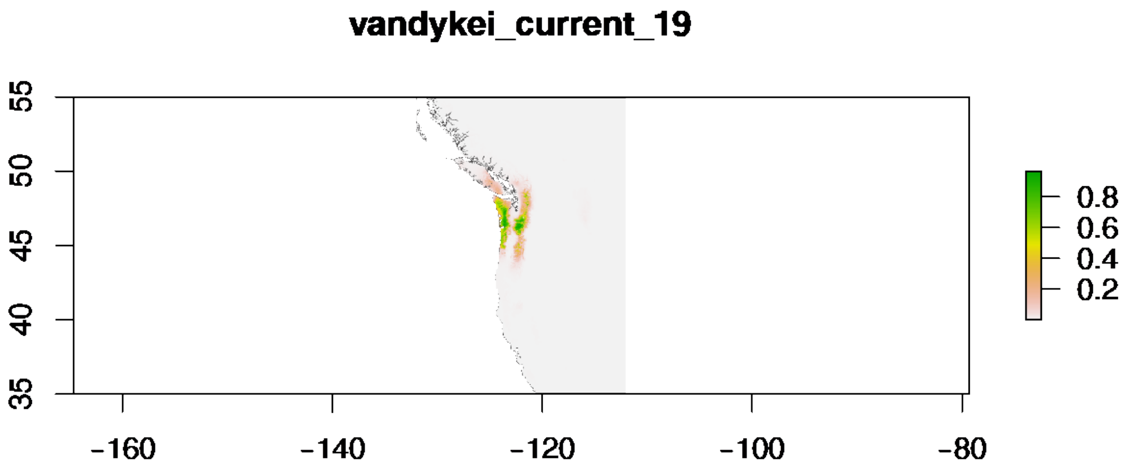

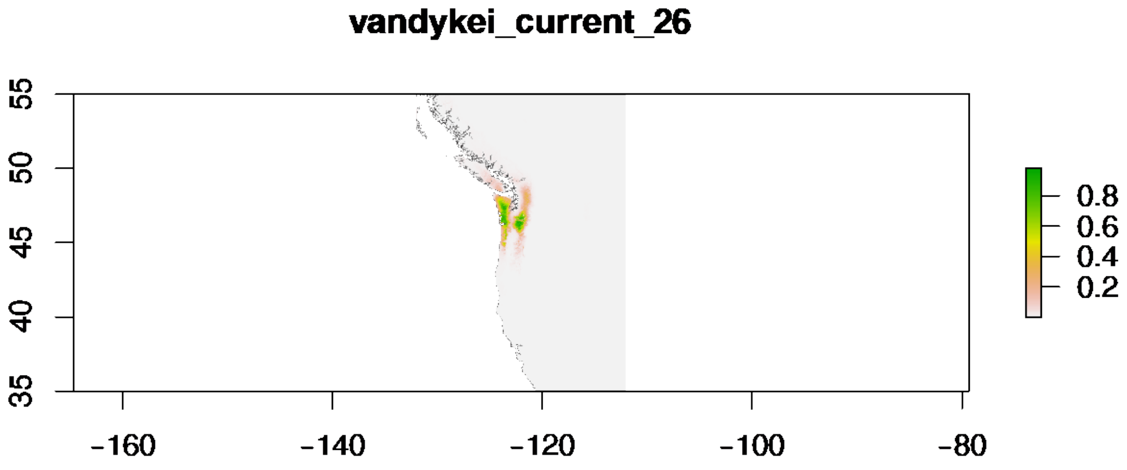

Figure S22: ***Plethodon vandykei*** current species distribution model before removal of correlated variables (all 19 bioclimatic (AUC= 0.991) variables and including elevation, solar radiation, wind speed, and water vapor pressure totaling 26 (AUC= 0.991)).

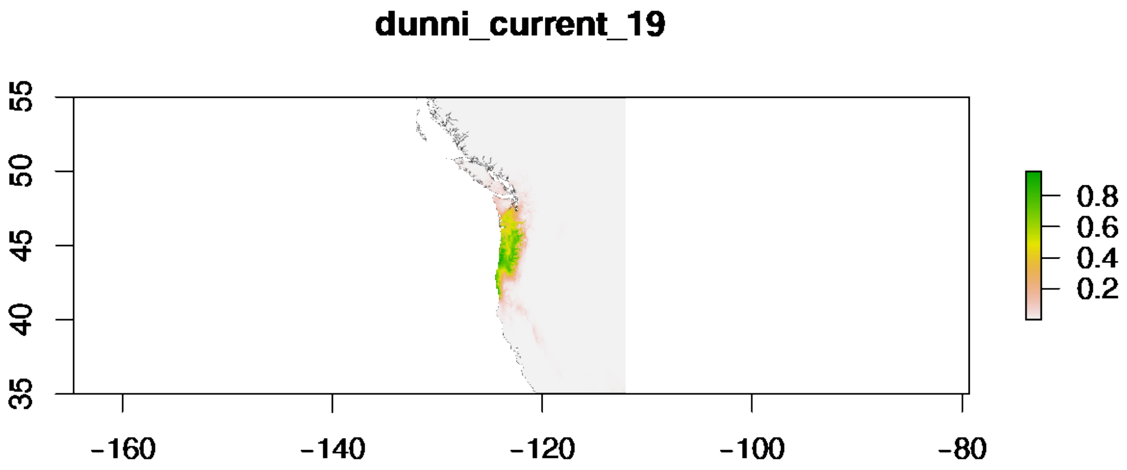

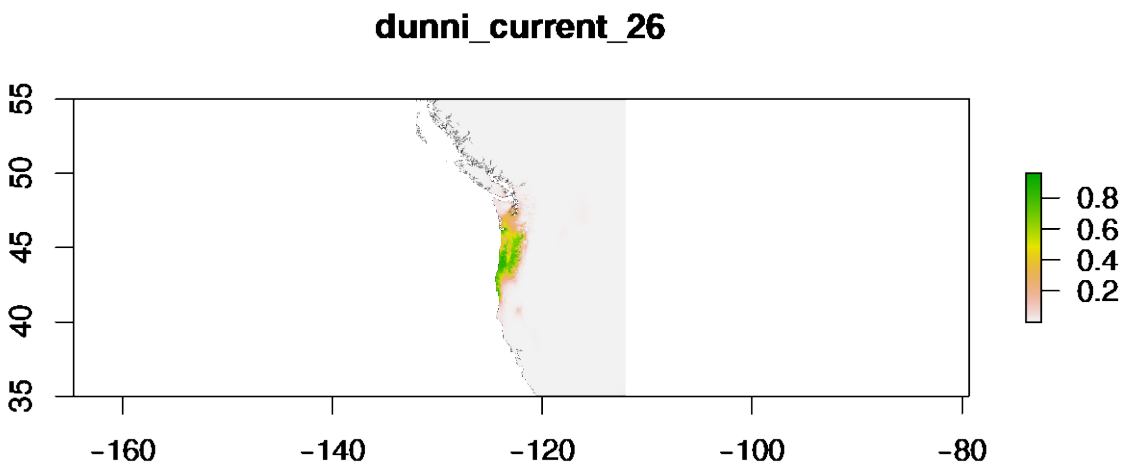

Figure S23: ***Plethodon dunni*** current species distribution model before removal of correlated variables (all 19 bioclimatic (AUC= 0.979) variables and including elevation, solar radiation, wind speed, and water vapor pressure totaling 26 (AUC= 0.981)).

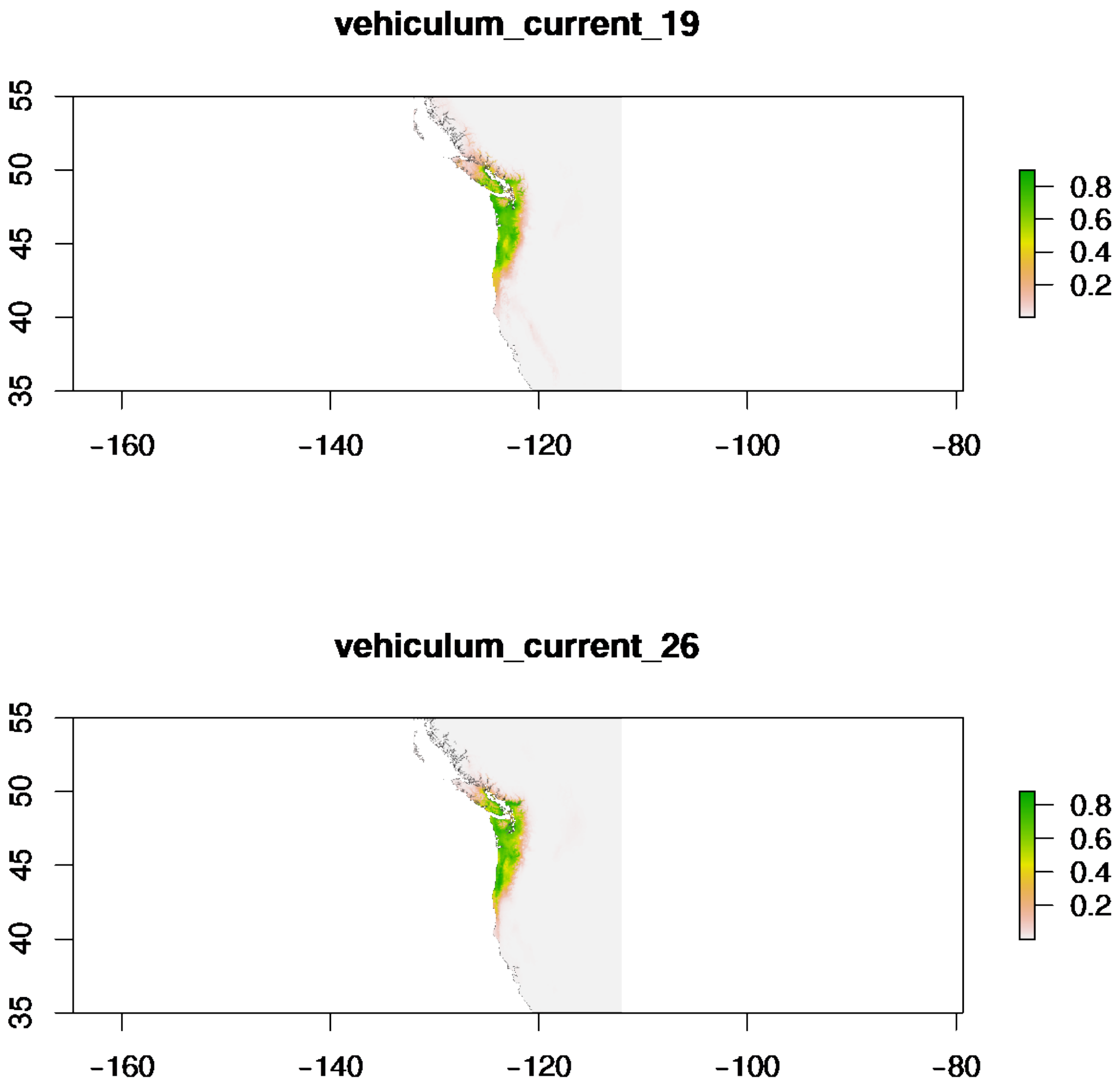

Figure S24: ***Plethodon vehiculum*** current species distribution model before removal of correlated variables (all 19 bioclimatic (AUC= 0.957) variables and including elevation, solar radiation, wind speed, and water vapor pressure totaling 26 (AUC= 0.956)).

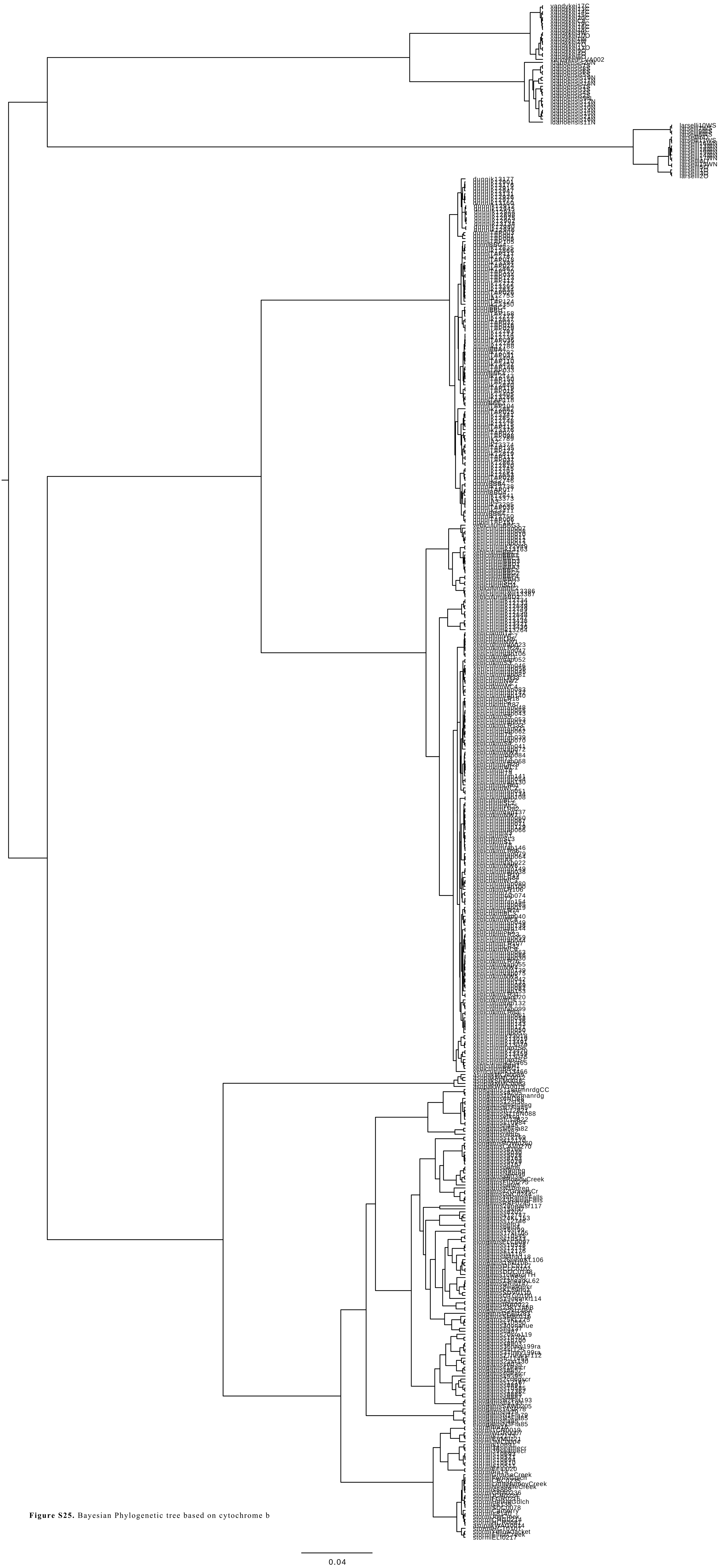

Figure S25. Bayesian Phylogenetic tree based on cytochrome b
